# Protocol for a pre-post, mixed-methods feasibility study of the Brain Bootcamp behaviour change intervention to promote healthy brain ageing in older adults

**DOI:** 10.1371/journal.pone.0272517

**Published:** 2022-11-29

**Authors:** Joyce Siette, Laura Dodds, Piers Dawes, Deborah Richards, Greg Savage, Paul Strutt, Kiran Ijaz, Carly Johnco, Viviana Wuthrich, Irene Heger, Kay Deckers, Sebastian Köhler, Christopher J. Armitage

**Affiliations:** 1 The MARCS Institute for Brain, Behaviour and Development, Western Sydney University, Westmead, New South Wales, Australia; 2 Centre for Health Systems and Safety Research, Australian Institute of Health Innovation, Macquarie University, Sydney, New South Wales, Australia; 3 School of Health and Rehabilitation Sciences, The University of Queensland, St Lucia, Queensland, Australia; 4 Department of Computing, Faculty of Science and Engineering, Macquarie University, Sydney, New South Wales, Australia; 5 Department of Psychology, Faculty of Medicine, Health & Human Sciences, Macquarie University, Sydney, New South Wales, Australia; 6 Centre for Ageing, Cognition and Wellbeing, Macquarie University, Sydney, New South Wales, Australia; 7 Department of Cognitive Science, Faculty of Medicine, Health & Human Sciences, Macquarie University, Sydney, New South Wales, Australia; 8 Centre for Health Informatics, Australian Institute of Health Innovation, Macquarie University, Sydney, New South Wales, Australia; 9 Department of Psychiatry and Neuropsychology, Alzheimer Centrum Limburg, School for Mental Health and Neuroscience, Maastricht University, Maastricht, The Netherlands; 10 Manchester Centre for Health Psychology, University of Manchester, Manchester, United Kingdom; 11 Manchester University NHS Foundation Trust, Manchester Academic Health Science Centre, Manchester, United Kingdom; 12 NIHR Greater Manchester Patient Safety Translational Research Centre, University of Manchester, Manchester, United Kingdom; UNITED KINGDOM

## Abstract

**Introduction:**

Behaviour change interventions represent key means for supporting healthy ageing and reducing dementia risk yet brief, scalable behaviour change interventions targeting dementia risk reduction in older adults is currently lacking. Here we describe the aims and design of the three-month Brain Bootcamp initiative that seeks to target multiple dementia risk and protective factors (healthy eating, physical, social and cognitive inactivity), through the use of multiple behaviour change techniques, including goal-setting for behaviour, information about health consequences and physical prompts to change behaviours that reduce dementia risk among older adults. Our secondary aim is to understand participants’ views of dementia prevention and explore the acceptability and integration of this campaign into daily life.

**Methods:**

Brain Bootcamp is a pre-post feasibility trial conducted in Sydney, Australia beginning in January 2021 until late August. Participants aged ≥65 years living independently in the community (n = 252), recruited through social media and flyers, will provide information about their demographics, medical history, alcohol consumption, smoking habits, mental health, physical activity, cognitive activity, and diet to generate a dementia risk profile at baseline and assess change therein at three-month follow-up. During the intervention, participants will receive a resource pack containing their individual risk profile, educational booklet on dementia risk factors and four physical items designed to prompt physical, social and mental activity, and better nutrition. Outcome measures include change in dementia risk scores, dementia awareness and motivation. A qualitative process evaluation will interview a sample of participants on the acceptability and feasibility of the intervention.

**Discussion:**

This will be the first short-term multi-domain intervention targeting dementia risk reduction in older adults. Findings will generate a new evidence base on how to best support efforts targeting lifestyle changes and to identify ways to optimise acceptability and effectiveness towards brain health for older adults.

**Trial registration number:**

ACTRN 381046 (registered 17/02/2021); Pre-results.

## Introduction

Dementia is one of the most common causes of disability, dependency and mortality among older adults and has considerable physical, psychological, social, and economic impacts on individuals diagnosed with dementia, their relatives, formal and informal caregivers, and society at large [1]. Cognitive ageing and dementia are global health priorities, with current estimates suggesting that 50 million people are living with dementia, with that estimate expected to triple by 2050 [2].

Despite extensive global research, there is no curative treatment for dementia available [3]. However, there is considerable scientific support for modifiable risk factors as contributors to dementia development in later life [4]. Recent estimations suggest that two in five dementia cases may be attributable to 12 common modifiable risk factors [5–7]. Previous research using population-attributable risk methods has further suggested that relative risk reductions of 10% per decade could result in reducing the prevalence of Alzheimer’s disease by 8.3% in 2050 [8]. Several health behaviours, such as regular physical exercise and high mental activity [9], have been associated with lower dementia risk in observational studies, and are receiving increasing attention in both research and policy [1, 10–14].

Importantly, in light of current knowledge about the complex and multifactorial etiology of dementia, targeting several risk factors and mechanisms simultaneously, as well as tailoring interventions to individual risk profiles, may be necessary to obtain optimal preventive effects [15]. So far, three large European multiple risk behaviour interventions trials on dementia risk have been completed: the Finnish Geriatric Intervention Study to Prevent Cognitive Impairment and Disability (FINGER; 1,260 participants aged 60–77 years) [16], the French Multidomain Alzheimer Preventive Trial (MAPT; 1,680 participants aged >70 years) [17], and the Dutch Prevention of Dementia by Intensive Vascular Care (preDIVA, 3,526 participants aged 70–78 years) [18]. The FINGER trial established a 2‐year intensive lifestyle and behaviour change intervention to successfully reduce cognitive decline across the span of 2 years in older adults [16]. Whilst exploratory subgroup analyses of MAPT and preDIVA also suggested cognitive benefits in subpopulations of participants with increased risk of dementia, these interventions did not affect cognitive performance or dementia risk. More recently, the US-based SPRINT‐MIND trial targeted maintaining acceptable blood pressure levels through an intensive anti-hypertensive treatment and found a decreased incidence of mild cognitive impairment or dementia in the intensive intervention (287 participants) versus standard treatment group (353 participants) [19, 20]. Lastly, the European HATICE trial used a coach-supported internet intervention to support individual self-management of cardiovascular risk and reported no change in secondary outcomes of cognition or dementia risk in 2,724 participants [15].

Whilst these studies indicate that administering multiple risk behaviour interventions to older at-risk adults show promise, they are often resource-intensive (e.g., FINGER trial required a concentrated 2 year intervention consisting of programs delivered by healthcare professionals on physical exercise (3–8 sessions), cognitive training (72 training sessions), as well as other clinical examinations to provide recommendations for lifestyle management [16]), and cannot be feasibly implemented in a wide-scale approach. Research priorities should therefore be targeted towards improving adherence, identifying individuals who may benefit, and extracting and repacking the active ingredients of a behaviour change interventions to reduce costs. Recent studies (e.g., Active Brains in the UK (study completion date 2027) [21], myCOACH in Australia (commenced August 2021) [22] and expansion of the FINGER trial to other countries [23, 24] are underway and show potential).

Innovative initiatives that are less time-intensive, target multiple risk behaviour changes, can be implemented at scale and enable individuals to successfully make and sustain changes in their daily routine, are required [8]. One potential approach is to adopt effective behaviour change principles in public health initiatives. Guided by the foundational principles of the Ottawa Charter for Health Promotion [18], our research team aimed to design and test a brief primary dementia prevention program, Brain Bootcamp, to reduce dementia risk. Whilst there is very limited evidence about which behaviour change techniques (BCTs) are effective at targeting dementia risk, reviews of BCTs within behaviour change interventions targeting modifiable dementia risk factors are available (e.g., physical activity and diet [25–28]). These reviews commonly found that goal-setting and feedback and monitoring of behaviour were associated with improved physical activity and healthy eating [29]. Further perspectives specific to dementia risk reduction also highlighted the need for trusted sources for education, availability of individual behaviour patterns and environments that enable individuals to take action whilst providing independence to establish, direct and maintain their progress [30]. Adopting both dementia risk reduction perspectives from the general population and synthesized BCTs in multiple modifiable risk factors, Brain Bootcamp is thus established on four core behaviour change domains of knowledge gain, goal setting, supportive environmental context and available social influences [31].

To target improved knowledge, an educational resource booklet was prepared by experts and will be used to promote dementia risk factor awareness, strengthen community action, create a supportive environment, and promote personal and social development for older adults. *Goal-setting* approaches focusing on four late-life dementia risk factors (physical inactivity, cognitive inactivity, social isolation, poor diets) are also included in the program and include both setting of a behaviour (e.g., I want to take 7,500 steps a day) and desired outcome (e.g., I want to improve my physical activity levels) for each risk factor.

The program also considered the *environmental context* and provides physical cues (e.g., pedometer) to prompt the participant to engage in the goal previously set. Finally, participants are provided with an individualized brain health risk score to provide information about social, health and environmental consequences on brain health. Overall, the project hopes to enable and motivate older adults to exercise more control over their brain health, and their environment especially during COVID-19 restrictions.

### Aim

By adopting behaviour change techniques, our primary aim is to evaluate whether a brief, lower-intensity and personally tailored program is able to lower dementia risk in older Australians. Our secondary aims are to explore whether the program can improve dementia awareness, and explore the feasibility and acceptability of the program and the research process.

## Materials and methods

### Study design

A prospective pre-post study will be used to evaluate the impact of the multiple behaviour change intervention on dementia risk. This pilot study will also test the feasibility, acceptability and usefulness of the Brain Bootcamp program, as well as the research protocol itself, and provide information on the usefulness of the research procedure through participant experiences. Mixed quantitative and qualitative methods will be employed in this 3 month pre- and post-test study. Ethical approval has been obtained with Macquarie University Ethics Research Committee (ref 52020917422563). Please see S1 File for the SPIRIT checklist and S2 File for the TIDieR checklist and S3 File for the original ethics protocol.

### Study population and recruitment

Participant recruitment began in January 2021 and continued until late August 2021. Inclusion criteria include community-dwelling individuals aged 65 years or more on date of consent, literate in English and who have access to the internet or can request a hard copy in order to complete the assessments. Exclusion criteria include individuals with a self-reported active episode of major depression and/or an existing diagnosis of dementia, inability to provide informed consent, or are currently enrolled in any behaviour change intervention.

### Participant selection and recruitment

**Fig 1:**
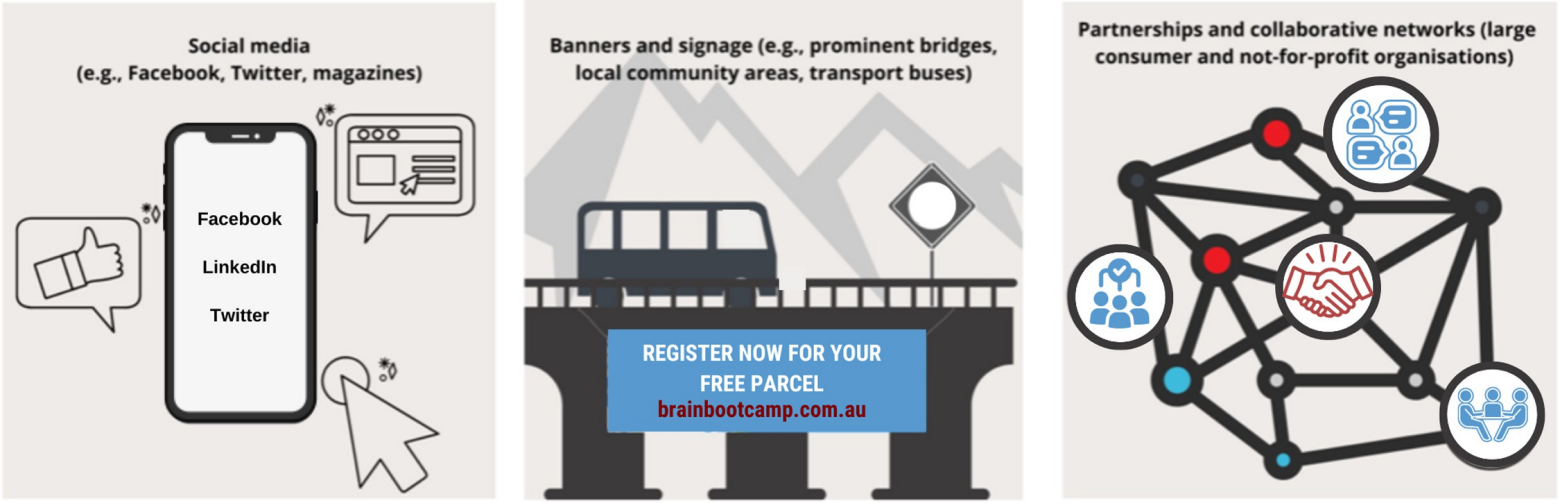
Graphical representation of the study’s advertising strategy (Source: Brain Bootcamp Research Team, Macquarie University, Australia).

Participants were consecutively recruited through a widespread advertising strategy, including standardised public advertisements and flyers disseminated at general medical practices, memory clinics, community newsletters, local print media, flyers and radio in New South Wales, Australia (**Fig 1**). Efforts were made to reach low socioeconomic status and minority individuals through e-newsletters and flyers of large organisations (e.g., Seniors Australia, Dementia Australia). Members of the general population who are interested in receiving a Brain Bootcamp Box are asked to register online (http://www.brainbootcamp.com.au) during the recruitment period by themselves or with the assistance of the research team. Upon registration they complete questions pertaining to the eligibility criteria and eligible participants provide informed written consent on the online registration form by ticking the category of their consent (e.g. “By clicking next you indicate that: You have read the Participant Information and Consent Form, You voluntarily agree to participate in this survey, You consent to having the research team contact you 3 months later, You are at least 65 years of age, You do not have a current diagnosis of dementia or severe depression”). For participants that do not have internet access or are otherwise unable to complete the online registration and survey, the research team either sends a postal version of the survey with replied paid postage or complete the survey over the telephone with the participant after obtaining informed verbal consent. Capacity to consent is measured in the online survey through several measures: (i) availability of consent documents (e.g., the Participant Information and Consent Form) which are embedded in the home page of the online survey, ensures that participants are provided with the requisite information about the study, have the opportunity to respond to queries by contacting the research team; and (ii) participants could not proceed unless the “I consent” button was pressed. Due to the eligibility criteria, it is assumed that participants had capacity to provide consent. This procedure was approved by the Macquarie University Ethics Research Committee (ref 52020917422563). To date, baseline survey data collection has been completed.

### Sample size

In order to calculate the target sample size, we used data from Deckers et al. [32]. Although the primary outcome, LIBRA scores, have mostly been used in proof-of-concept trials, Deckers et al. [32] found in the FINGER trial a mean difference in LIBRA index scores between baseline and after 12 months of 0.6 point in the intervention group. Recent evidence further simulated “small,” “medium,” and “large” intervention effects between-group differences of the LIBRA index as −0.19, −0.31, and −0.52 respectively [33]. To observe a large interventional effect in our study, we will require a sample size of 176 participants, using a 90% statistical power and a 5% maximum probability of type 1 error alpha. This sample size was calculated using the t-test package of the Rstudio software (power.t.test) and was rounded to 252 to account for a possible 30% attrition rate.

The anticipated target sample size for semi-structured interviews is 60 participants. This is to ensure the evaluation accounts for variables that may influence recruitment, adherence and overall impact of the intervention. These include brain risk score (high and low), age (65–74 years and ≥75 years), gender (male and female), education (≤12 and >12 years) and socioeconomic status (high and low) and locality (rural and metropolitan) with a minimum of 10 participants per variable. Interviews with participants will continue until data saturation is reached for each of those categories (typically around 12 participants per group [34]).

### Procedure

**Fig 2:**
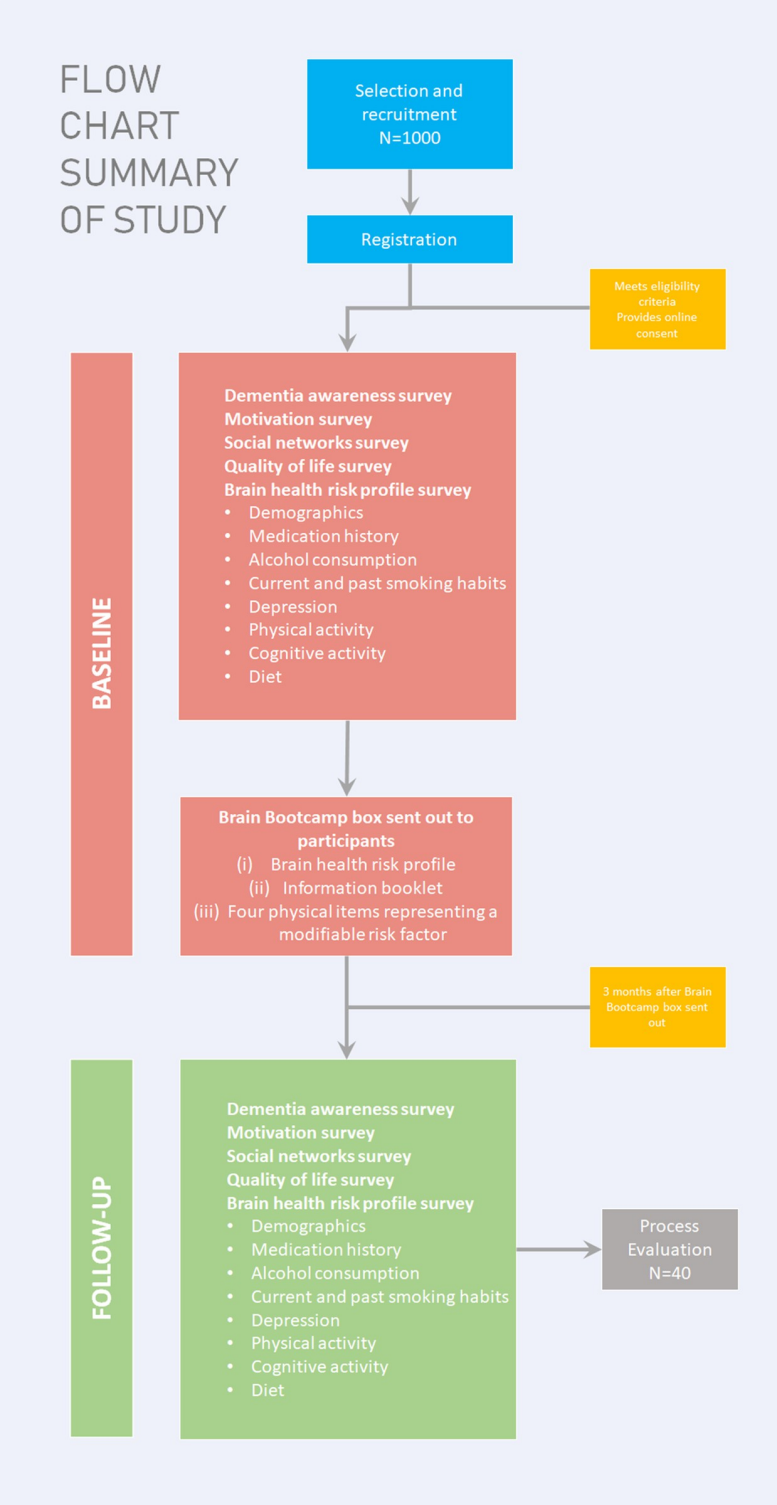
Flow chart summary of study.

Participants will visit our website (http://www.brainbootcamp.com.au) to register and complete online assessments on dementia risk literacy and to create a brain health risk profile, identified using the ‘‘LIfestyle for BRAin health” (LIBRA) index, which is shown to be a valid predictor of the development of dementia and cognitive impairment [35–39]. The index is a weighted compound score of the presence/absence of 12 modifiable risk and protective factors (i.e., hypertension, obesity, high cholesterol, diabetes, coronary heart disease, chronic kidney disease, physical inactivity, alcohol intake, smoking, depression, diet and cognitive activity), that can be targeted by lifestyle interventions and vascular risk management in primary care. LIBRA ranges from −5.9 to +12.7, with higher scores indicating greater dementia risk [40]. Scoring details for individual risk factor weights and calculation of the algorithm are available from Deckers et al. [41]. The LIBRA index has been developed as an instrument to show an individual’s potential for dementia prevention based on epidemiological evidence for dementia risk factors and expert Delphi consensus [42] and has been validated against large, international longitudinal cohort studies [40]. The researchers have identified that a one-point increase in the LIBRA index is associated with a 19% higher risk for dementia and 9% higher risk for cognitive impairment for older adults [35]. The index has also been shown to be a suitable intermediate outcome for trials [33]. Data will be collected on the following variables at baseline and three months later from all participants (**Fig 2**). All individual instruments have been validated for self-report and demonstrate ability to detect change over time [39, 43–52].

### Measures

- Demographics including age, gender, income, marital status, living arrangements, ethnic background, current or previous occupation, education level (as identified by completed years of schooling and tertiary studies)
- LIBRA Index [29] will be assessed using below sub-measures:
  - Medical history (i.e., self-reported presence of current cardiovascular disease, diabetes mellitus, chronic kidney disease, hypertension, hypercholesterolemia, body mass index (weight and height)).
  - Alcohol consumption (current daily average alcohol consumption, 0 or up to and including 1 glass per day or more than 1 glass per day).
  - Current and past smoking habits (i.e. current smoker, yes or no).
  - Current depressive symptoms using the 9-item brief depression severity measure, Patient Health Questionnaire (PHQ-9). Responses range from 0 (“not at all”) to 3 (“nearly every day”) [53]. It has been used in several contexts including medical settings and amongst the general population [43] and has a sensitivity and specificity of 88% for major depression [53]. A score 10 or above indicates symptoms of moderate, moderately severe and severe depression [27].
  - Levels of current physical activity will be measured with a custom-made item developed by the research team and used in the short version of the LIBRA index [25]. It consists of one question “Do you generally consider yourself as an active person, who regularly performs exercise in which you start to sweat lightly?” with three possible responses “Yes, A little, and No”.
  - Cognitive activity will be measured using the Cognitive Reserve questionnaire (CRIq) [49]. This 20 item instrument measures cognitive activity across three sections; education (e.g. completed years of education, training courses), previous working activity (e.g. adulthood professions) and current leisure time (e.g. reading newspaper, playing music, social and physical activities) [49]. Performance below the cut-off in at least 2 sections indicates a higher risk of cognitive impairment [54]. This instrument has established construct validity in healthy populations.
  - Current adherence to diet will be measured using the Mediterranean Diet Adherence Screener (MEDAS) [50]. This is a validated 14-item scale with a score of 0 or 1 assigned to each item [55]. A maximum score of 14 indicates the greatest adherence to the diet.
- Social networks: will be measured using the 6-item version of the Lubben Social Network Scale (LSNS-6) and refer to the previous month of social contact. Internal reliability (0.83) is good and the two sub-scales (family and friendships) both demonstrate high levels of internal consistency. Scores range from 0–30 with higher scores indicating more social engagement and scores <12 identifying individuals at risk of social isolation [44].
- Quality of life: will be measured using the EQ-5D-5L scale for the present day. It consists of a self-administered health index and a 100-point visual analogue scale (VAS), for participants to rate their current health state from 0 as their ‘worst imaginable health state’ to 100, their ‘best imaginable health state’ [56]. The instrument covers mobility, self-care, pain/discomfort, usual activities and anxiety/depression [57]. Utility scores are quantified along a continuum that ranged from -0.59 (worst health) to 1.00 (perfect health). It has demonstrated convergent validity [45] and good reliability for individuals with diverse health conditions [46, 47].
- Motivation: Motivational attitudes and beliefs to modify lifestyle specific to dementia risk reduction will be measured using Motivation to Change Lifestyle and Health Behaviour for Dementia Risk Reduction (MCLHB-DRR) [58]. This is a 27-item tool, commonly used in the evaluation of dementia prevention programs. It has been validated amongst individuals aged 50 and over with moderate to high internal reliability and test-retest reliability. Additional questions on individual capabilities and opportunities will also be asked.
- Dementia awareness: Dementia awareness and literacy will be measured with a previously used questionnaire adapted from the *MijnBreincoach* public health campaign [51]. This 22-item questionnaire includes ten items from the UK’s British Social Attitudes (BSA) survey [52], covering self-reported knowledge of dementia, general statement on the possibility of dementia risk reduction, personal experience with individuals with dementia, dementia risk awareness and knowledge of 14 modifiable dementia risk and protective factors (i.e., hypertension, smoking, physical activity, depression, diabetes, obesity, coronary heart disease, chronic kidney disease, hypercholesterolemia, mental activity, low to moderate alcohol intake, healthy diet, midlife hearing loss and social connections) [39, 59]. In addition, three sham factors will be included (use of painkillers, personal hygiene and having children) to check for monotone answering tendency. Additional items will ask for other factors respondents consider to be related to dementia risk motivation. Participants will be asked to what extent they agree or disagree on a 5-point Likert scale ranging from ‘strongly disagree’ to ‘strongly agree’.
- Impact evaluation survey (follow-up only): 48 items on five domains including (i) overall experience (e.g., participants will be asked to what extent they agree or disagree on a 5-point Likert scale ranging from ‘strongly disagree’ to ‘strongly agree’ to statements such as ‘Brain Bootcamp increased my awareness about dementia risk factors’, as well as open text responses to questions on what they liked and disliked about the program and recommendations for the future), (ii) recognition of the program’s call to action phrases e.g., ‘Stay curious’ and ‘Exercise regularly’, (iii) item use and preference (e.g., frequency of use, preference order) and (iv) evaluation of impact of program on each risk factor (e.g., whether a goal was set, what the goal was, whether the participant was able to meet their set goal and how did the item support goal achievement).

A detailed summary of the outcome measures and assessments used is provided in **Table 1**. Further exploration of these outcomes and measures will also be addressed in the qualitative interviews (see Impact Evaluation section).

**Table 1:** Summary of outcomes and their measures.

Outcome		Data collection instrument	Time
**Primary Outcome**			
**Dementia risk**		LIBRA index [35]	T1, T2
**Secondary Outcomes**			
**Dementia Awareness**	Adapted questionnaire [51, 52]		T1, T2
**Motivation**	Motivation to Change Lifestyle and Health Behaviour for Dementia Risk Reduction (MCLHB-DRR) [58]		T1, T2
**Social networks**	Lubben Social Network Scale (LSNS-6) [44]		T1, T2
**Quality of life**	EuroQol Group EQ-5D-5L [56]		T1, T2
**Overall experience**	Adapted evaluation survey [60] and impact evaluation interviews		T2

### Intervention

**Fig 3:**
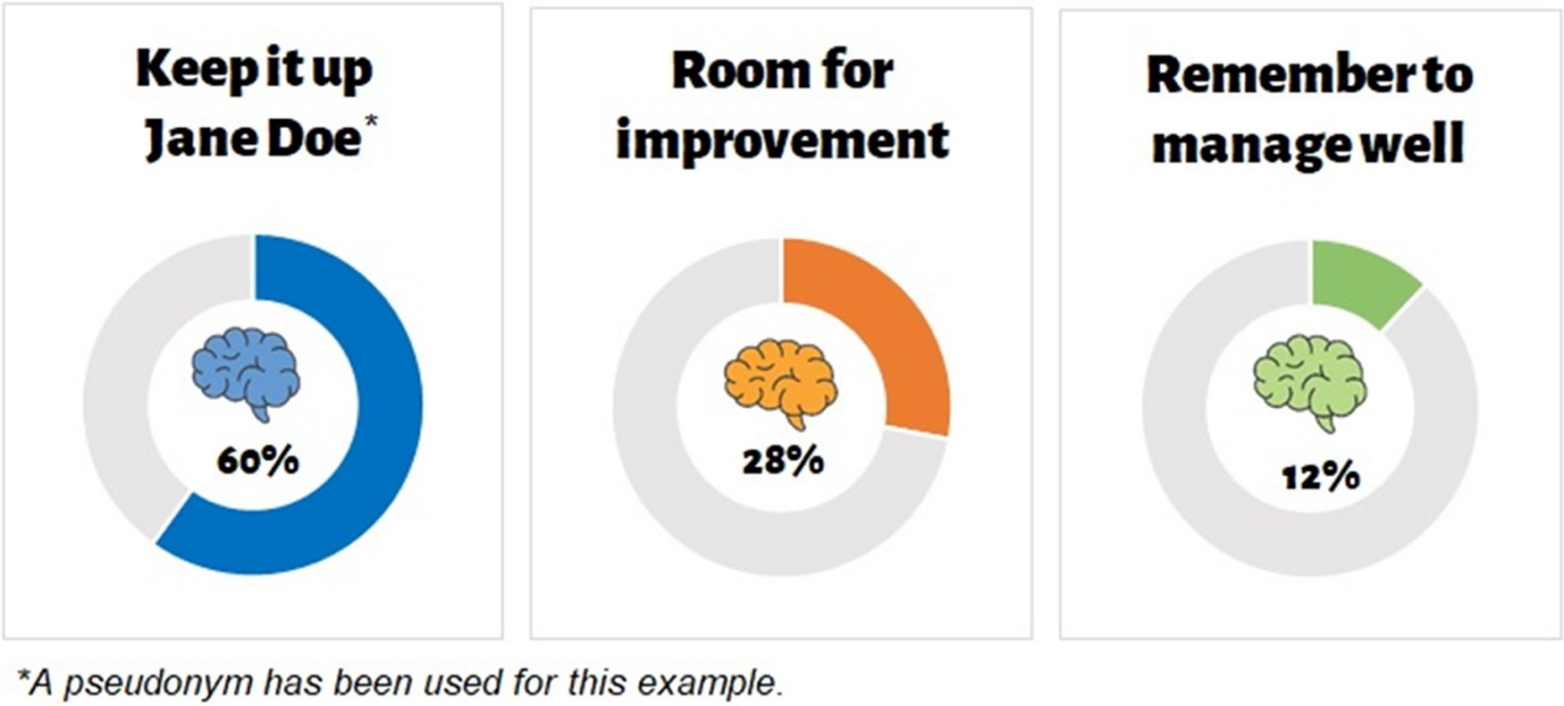
Example of a personalised brain health risk profile.

Upon completion of the baseline surveys, participants will receive a Brain Bootcamp Box via registered post which contains items that aim to use the four behaviour change techniques of goal setting and planning, shaping knowledge, environmental context and social influences [25–28] to change multiple health behaviours. These techniques have been chosen through reiterative discussions with the research team and consumer stakeholders and contain: (i) personalised information on their brain health risk profile describing areas where they are doing well in terms of preserving their brain health as well as areas they can make sustainable changes to their lifestyle to reduce their risk and areas which they need to ensure they manage well (BCT comparison of outcome) (**Fig 3**); (ii) an information booklet about dementia literacy and associated health-related behaviours such as advice on healthy eating, and being physically, socially and mentally active (BCT knowledge gain); and (iii) four physical items that they can use to initiate healthy brain habits (BCTs supportive environmental context and goal setting) [61].

Each item further represents a modifiable risk factor (e.g. olive oil and balsamic vinegar to target diet, pedometer to target physical activity, social calendar containing available social events to address social networks, and brain flash cards to stimulate cognitive activity). Participants can use any of these items in their daily lives to remind them of the importance of brain health and the simple changes they can make in their own daily lives to reduce their risk of dementia and cognitive decline.

First, participants are provided with a personalised brain health profile and educational booklet which aims to inform the individual of their own risk of dementia, which provides feedback on specific behaviours that are contributing to better or poorer brain health. The wording of feedback underwent iterative development between clinicians, investigators and original developers of the LIBRA index to ensure phrasing was acceptable and accessible. Suggestions for change are completed through a goal setting approach where individual goals are developed by the participant based on their brain health profile and proposed suggestions in their profile. Participants are encouraged to write their own goals in the educational booklet and monitor their progress in their social calendar.

Similarly, items such as the pedometer, olive oil and balsamic vinegar, and brain flash cards provide an opportunity for individuals to adopt healthy behaviours (for instance, to substitute their regular cooking ingredients with brain healthy oils, encourage the individual to repeatedly engage in cognitive stimulation and physical activity). Brain flash cards include four different topics, including brain teasers (e.g., “Say the months of the year in reverse order”), problem solving (e.g., “Select the word that does not belong: Canberra, Madrid, Ottawa, Cairo, Colombo”), brain games (e.g. “Look around you and within two minutes try to find 5 red things that will fit in your pockets, and 5 blue objects that are too big to fit”), and learning new languages (e.g., common phrases in Spanish, Arabic, Chinese, Italian, Greek, Tagalog, Hindi, French, German and Japanese).

The educational booklet was drafted by a team experienced in health psychology, clinical psychology, neuropsychology, public health, audiology and health informatics and was based on the existing prevention research [5] and shared with members of the public to further validate the content.

Patient and public involvement was sought during the development of this study to inform the objects and items in the box. Discussions were held with two co-investigators with lived experience and aged care organisations providing care to older adults residing in the community, using laypersons’ language to enable common understanding and encourage their feedback. Items in the box (including physical items, educational booklet and the personalised brain health profile) were revised and refined to focus on aspects that the public were most vocal about.

Approximately six weeks after the participant has received the box, the research team will contact the participant to inform them that they are halfway through the intervention period and to encourage use of the box items and resources if they have not started yet. Participants are encouraged to reach out to the research team for any further assistance.

### Impact evaluation

An impact evaluation will be conducted as part of the trial to understand the experience of participants involved in the initiative and the feasibility of the intervention. At the end of the trial period, a theoretical sampling approach will be used to invite participants (N = 60) based on their brain risk score (high and low), age, gender (male and female), education, locality (rural/regional and metropolitan) and socioeconomic status (high and low) for semi-structured interviews. Interviews will explore in-depth views of the information they received from their profile, whether they have accessed the resources in their Brain Bootcamp pack, any health behaviour change goals, how they did/did not incorporate recommendations into their daily life, and their perception of the overall impact of the initiative. We will also investigate why participants did not complete the trial by including a sample of those who dropped out. The evaluation will be based on these overarching questions from the Australian Government impact evaluation framework [60]:

- Did the initiative make a difference?
- How much of a difference did the initiative make?
- For whom, in what situations, and in what ways did the initiative make a difference?
- To what extent can a specific impact be attributed to the initiative? (e.g., Was there a particular technique that you thought was most useful for modifying your behaviour?)
- How did the initiative make a difference? (e.g., How did the program support your awareness of dementia risk factors?)
- Will the initiative work elsewhere? (e.g. What is needed for the initiative to work elsewhere and for our recruitment processes to successfully identify other population groups? i.e., in aged care, for rural locations, for participants from culturally and linguistically diverse backgrounds?)
- What is needed for the initiative to work elsewhere? (e.g. Please tell us what changes could be made to improve Brain Bootcamp? How can we ensure follow-up data is collected?)
- Were the chosen measures appropriate? (e.g., acceptability of survey length, survey questions, display of brain health profile)

Other process evaluation data will include drop-out rate at follow-up, data analytics of the website and reporting of adverse outcomes. At mid-point of the intervention, we will contact all participants to identify issues, whereby these insights will provide the research team with ongoing information on participants’ experience and engagement levels. See S1 Fig for our logic model.

### Data management and analysis plan

Data will be collected, managed and analysed throughout the project according to our ethics project description. Interview scripts and questionnaire data will be managed and stored on the secure servers at Macquarie University. Participant survey data will be treated in a strictly confidential way and analysed in anonymous form. All identifiable information in the participant interviews (e.g., names, locations) will be removed prior to analysis.

All surveys will be visually inspected for completeness on collection to minimise missing data. Assessment data will be entered into databases and analysed using standard statistical software (eg, SAS V.9.2 or SPSS V.27). Quantitative data obtained in response to structured assessment items will be entered, cleaned, analysed and summarised.

Descriptive statistics will be computed for participant demographics and all components of the LIBRA index score. Where appropriate, multiple imputation will be used to manage missing data and account for potential attrition bias. Pre-post difference in outcome scores including the LIBRA index, dementia awareness, social networks and quality of life will be analysed using dependent sample t-tests and χ2 as appropriate. Dependent sample t-tests and one-way analysis of variance (ANOVA) will be used to examine differences in baseline LIBRA scores among demographic (e.g., age, gender, socioeconomic status) and service (e.g., receiving aged care services) factors.

To explore whether the program impacted on dementia risk, descriptive analyses will be first used to quantify changes in participants’ LIBRA index scores at 3-month follow-up. Multilevel modelling techniques will then be used to determine whether higher levels of engagement in the program (e.g., whether a goal was set and achieved) is associated with a change in LIBRA index score. As change in LIBRA index scores is normally distributed [32], modelling of a random effects structure will aid a more exact inference about observed effects, whilst allowing for the estimating of variance of the LIBRA index within the group [62].

We will first screen for covariates using the univariate association between the LIBRA index and core socio-demographic variables (gender, age, country of birth), followed by psychosocial variables (quality of life, social networks) selecting those with p<0.2 and adjust for baseline health characteristics. The final stage will add interaction terms between groups such as age (grouped 65–74, 75–84 and 85+), race, and years of education (<12 vs ≥12) that predicts change in dementia awareness scores. Interaction terms will be retained in the final model only if they are significant. We will enter into the model only those covariates that are not multicollinear based on the variance inflation factor criterion [63].

Semi-structured interviews will be audio recorded and transcribed. Interview data obtained in the process evaluations will be analysed following the appropriate qualitative approach (e.g., phenomenology or narrative research) for content and themes that emerge, and coded and categorised using N Vivo software between a minimum of two researchers. Qualitative data analysis will involve an initial open coding of all transcriptions, followed by axial coding using grounded theory techniques [13] whereby initial codes, indicators and concepts are triangulated with other findings (e.g., quantitative) leading to refined analytical levels, relevant to the study aims. Analysis will be carried out thematically and iteratively using a constant comparative approach. A coding frame will be developed based on codes arising from the first 10 transcripts by two independent researchers and refined. The research team will continue to code all transcripts and further refine codes based on subsequent transcripts and discussions with the research team.

### Ethical considerations and declarations

This project will comply with the principles of the Declaration of Helsinki for Human Rights and will be overseen by the Macquarie University Human Research Ethics Committee, who has approved the study protocol (ref 52020917422563). None of the assessments or procedures are expected, or known, to cause significant harm. The feedback that is provided to participants has undergone extensive review and is not expected to cause any undue anxiety, however a phone number and email to contact Brain Bootcamp researchers is accessible and available in the box and on the Brain Bootcamp website. Participants are encouraged to contact researchers if they would like further clarification of their brain health profiles or other program procedures. Participants will also be free to discontinue involvement at any time if they wish. Protocol modifications (e.g. changes to eligibility criteria, outcomes, analyses) will be submitted to the relevant parties including investigators, ethics committee and trial registries and receive approval before commencing proposed modifications.

Written informed consent will be obtained for all participants. Some participants may be in the mild stages of cognitive decline, and therefore would generally be expected to be able to provide informed consent. In instances where the participant’s level of impairment increases, such that they are no longer able to provide informed consent, the provisions of the Mental Capacity Act will be followed. Participants will be informed of their right to withdraw at any time without their care being affected in any way. Furthermore, as the study is dealing with individuals who may have increased risk of depression or cognitive decline, treating GPs will receive clinically relevant data whenever appropriate. Such a procedure is noted in the participant information and consent form.

### Status and timeline of the study

Baseline data collection is currently complete. Follow-up data collection commenced in April and is ongoing. Semi-structured interviews took place between July to September 2021. Data analyses for both qualitative and quantitative components are anticipated to commence in July and continue until November 2021. See S1 Fig for the study’s program logic.

## Discussion

The Brain Bootcamp program, to our knowledge, is the first brief multi-domain intervention comprising of multiple behaviour health strategies and physical prompts targeting behaviour change towards four dementia risk factors for older adults. Various components were made accessible and available (e.g., online survey to generate dementia risk, delivery of box to support initial and ongoing goal setting) to facilitate participant engagement in simple strategies to benefit brain health. This study will address an important gap by evaluating a short-term intervention addressing four modifiable dementia risk factors, including social connectivity, which is an emerging risk factor that has not been explored in-depth [5]. Our study will further examine how the program can be delivered effectively in the community, and determine useful approaches to reaching disadvantaged groups, as well as the feasibility, uptake and efficacy of the intervention from the perspective of older adults. We intend to use our mixed-methods findings to further optimize and refine the intervention to ensure its suitability, acceptability, and feasibility for sub-groups.

Although prevention of dementia and late-life cognitive decline is a major public health priority, there are currently no established prevention models for implementing such strategies into policy and practice [64]. Given the lack of evidence regarding low-cost interventions targeting reductions in dementia risk, this work, if found to be effective and feasible, may contribute to risk reduction guidelines at a public health policy and healthcare system level in several ways. First, by providing tailored dementia risk scores and subsequent suggested behaviours for specific individuals, our results will validate the acceptability of a ‘precision risk reduction approach’ [65] to support targeting dementia risk. Second, using the validated LIBRA index to detect at-risk individuals can further facilitate precision risk reduction as well as demonstrate the index’s use in future prevention trials.

Third, our results will further inform the efficacy of our combination of BCTs. Although BCTs are most often used as part of a public health intervention, the effectiveness of a single BCT, an arrangement of BCTs or a specific number of BCTs remains largely unknown [66]. Michie et al. [67] identified that blending the BCT of ‘self-monitoring’ (e.g., goal setting, monitoring of behaviour, and receiving feedback on behaviour) with one or more other BCTs was associated with healthier behaviour of eating and physical activity. Our results will further guide future research to explore the effectiveness of single or arranged BCTs for future interventions targeting dementia risk reduction.

### Strengths and limitations

Our study has some limitations. Firstly, the study is limited due to the confines of the study design that prevented true randomization and lacked a control group, which limit our understanding of causality and intervention effectiveness. Secondly, results from this study only pertain to an Australian population, and are not generalizable to other populations, and use self-reported data of medical and physical health conditions. Thirdly, participant recruitment is conducted via a non-random recruitment strategy which may lead to potential confounders and risk of bias in the interpretation of the results. We have developed a recruitment strategy targeted at a sample that represents the 65 years and older population in terms of gender and educational levels, however a moderate level of reading and understanding English (Year 7 level) is required for participation. Such inclusion criteria means that a large proportion of Australia’s multicultural and linguistically diverse individuals may be excluded, and key perspectives regarding dementia risk reduction programs are under-represented. Indeed, individuals from culturally and linguistically diverse (CALD) backgrounds are often excluded from dementia research studies due to language barriers, causing gaps in the evidence base [68]. Research has further shown that CALD older adults have poorer dementia literacy, more negative attitudes about dementia, and present later for diagnosis [69] and face different participation and economic barriers, compared to Australian-born individuals [70]. Future programs thus need to be more culturally-diverse and culturally-inclusion for older adults of different ethnic backgrounds.

Although the above limitations can be overcome by a random sampling approach coupled with multi-lingual communication, including literacy and health literacy considerations to enable fair recruitment, it was unfortunately not feasible in the present study. If findings from this study demonstrate effectiveness and acceptability, the research group intends to proceed with both a randomized control trial to further explore efficacy using a control group, and a design-based approach containing mixed-methods to understand enablers and barriers, behaviours and contextual elements that are relevant to dementia prevention for CALD populations.

The study’s strengths include the extensive data collected that will provide valuable insight to the effects of a short-term program within a healthy population of Australian older adults. Findings will inform on the complex interaction between dementia risk, motivation to change, dementia literacy, and psychosocial outcomes of social networks and well-being, that may support the employment of short-term programs as a tool for future dementia prevention interventions.

### Dissemination

Results of the study will be disseminated to the scientific, medical and general public and on our website (http://www.brainbootcamp.com.au). In addition, a summary of aggregate results will be produced and sent to participants who engaged in the study. Results will be published in peer-reviewed international journals and will be presented at national and international conferences and symposiums. In addition, results and their practical implications will be disseminated with not-for-profit organisations, government bodies, and aged care settings.

## Conclusion

The study will provide an initial insight into how short-term initiatives targeting improved literacy on dementia risk and protective factors can be successfully conducted. Using a mixed-methods approach, this study will provide a detailed understanding of the factors supporting risk reduction behaviours and how to better drive dementia risk literacy. It will further assist in targeting individuals who are most likely to benefit from protective factors, therefore guiding optimal clinical care and follow-up procedures.

## Supporting information

S1 File SPIRIT schedule of enrolment, intervention, and assessments. (PDF)

S2 File TIDier checklist. (DOCX)

S3 File Ethics protocol. (PDF)

S1 Fig Program logic. (PNG)
