# Supplementary material for: Protocol for a pre-post, mixed-methods feasibility study of the Brain Bootcamp behaviour change intervention to promote healthy brain ageing in older adults
Source: PLoS One. 2022 Nov 29;17(11):e0272517. doi: 10.1371/journal.pone.0272517 (PMC9707764; doi:10.1371/journal.pone.0272517)
Supplement: S2 File — (DOCX) [file pone.0272517.s002.docx]

Macquarie University

australian institute of health innovation

*Centre for Health Systems and Safety Research*

**Project Description**

**Title: Brain Bootcamp: an initiative targeting dementia awareness and risk among older adults**

**Short Title: Brain Bootcamp**

**Version #: 1**

**Protocol Date: 07/10/2020**

**Ethics Statement:**

The study will be conducted in accordance with the *National Statement on Ethical Conduct in Human Research* (2007), the *CPMP/ICH Note for Guidance on Good Clinical Practice* and consistent with the principles that have their origin in the Declaration of Helsinki. Compliance with these standards provides assurance that the rights, safety and well-being of trial participants are respected.

Contents

[Contents 1](#_Toc52974439)

[Summary 3](#_Toc52974440)

[Project Team Roles and Responsibilities 7](#_Toc52974441)

[1. BACKGROUND AND INTRODUCTION 10](#_Toc52974442)

[1.1. Literature Review 10](#_Toc52974443)

[2. HYPOTHESIS 11](#_Toc52974444)

[3. STUDY OBJECTIVES 12](#_Toc52974445)

[3.1. STUDY 1 PRIMARY OBJECTIVES 12](#_Toc52974446)

[3.2. STUDY 2 PRIMARY OBJECTIVE 12](#_Toc52974447)

[3.3. STUDY 3 PRIMARY OBJECTIVES 12](#_Toc52974448)

[4. Project DESIGN 12](#_Toc52974449)

[4.1. DESIGN 12](#_Toc52974450)

[4.2. EXPECTED PARTICIPANT NUMBERS 12](#_Toc52974451)

[4.3. SETTINGS 13](#_Toc52974452)

[5. STUDY PARTICIPANTS 13](#_Toc52974453)

[5.1. INCLUSION AND EXLUSION CRITERIA 13](#_Toc52974454)

[6. STUDY PROCEDURES 14](#_Toc52974455)

[6.2. STUDY PLAN 14](#_Toc52974456)

[6.3. PARTICIPANT RECRUITMENT AND SCREENING 15](#_Toc52974457)

[6.4. PARTICIPANT ENROLMENT 17](#_Toc52974458)

[6.5. INFORMATION AND CONSENT 17](#_Toc52974459)

[6.6. DATA COLLECTION 19](#_Toc52974460)

[6.7. END OF STUDY WITHDRAWAL PROCEDURE 20](#_Toc52974461)

[6.8. PATIENT WITHDRAWAL 20](#_Toc52974462)

[7. OUTCOME MEASURES 20](#_Toc52974463)

[8. STATISTICAL CONSIDERATIONS 25](#_Toc52974464)

[8.1. SAMPLE SIZE OR POWER CALCULATION 25](#_Toc52974465)

[8.2. PROVIDE A DETAILED ANALYSIS PLAN 25](#_Toc52974466)

[9. QUALITY CONTROL AND ASSURANCE 26](#_Toc52974467)

[9.1. CONTROL OF DATA CONSISTENCY 26](#_Toc52974468)

[9.2. AUDITS 26](#_Toc52974469)

[9.3. PROTOCOL AMENDMENTS 26](#_Toc52974470)

[10. ETHICS 26](#_Toc52974471)

[10.1. INVESTIGATOR AUTHORISATION PROCEDURE 26](#_Toc52974472)

[10.2. PATIENT PROTECTION 26](#_Toc52974473)

[11. CONFIDENTIALITY AND STORAGE AND ARCHIVING OF STUDY 27](#_Toc52974474)

[12. RESULTS, OUTCOMES AND FUTURE PLANS 27](#_Toc52974475)

[13. TRIAL SPONSORSHIP AND FINANCING 28](#_Toc52974476)

[14. REFERENCES 29](#_Toc52974477)

## Summary

**Study title:** Brain Bootcamp: an initiative targeting dementia awareness and risk among older adults

**Protocol version** 1

**Overview** This project will be made up of 3 studies that aim to evaluate the impact of the public health campaign in improving dementia risk and dementia awareness, understand the facilitating factors to making and sustaining lifestyle changes, and to assess the acceptability and validity of a new cognitive assessment for older adults. These are described in detail below.

**Objectives**

Study 1 (i) To evaluate the impact of the Brain Bootcamp campaign in increasing dementia awareness and knowledge of modifiable lifestyle factors in older Australians

(ii) To evaluate the impact of the brain bootcamp campaign in improving older adults’ brain health risk profile

Study 2 To understand participants’ views of dementia prevention and the adoption of brain bootcamp into their daily lives

Study 3 (i) To determine the acceptability and feasibility of new virtual reality technology in assessing cognitive function in older adults

(ii) To assess the ability of the virtual reality technology to discriminate between cognitively intact and cognitively impaired participants

**Project design** **Mixed methods**

Study 1 Prospective pre post study

Study 2 Qualitative study

Study 3 Cross-sectional feasibility study

**Planned Sample Size**

Study 1 500 community-dwelling older adults residing in NSW

Study 2 40 older adults from Study 1 participants

Study 3 100 older adults (aged 65+) and 100 young adults (aged 18+)

**Selection criteria**

Study 1 and Study 2 Inclusion criteria

Population-based sample of community-dwelling adults aged over 65 years old

Exclusion criteria

Individuals with an active episode of major depression, an existing diagnosis of dementia, inability to provide informed consent, or are currently enrolled in any lifestyle change intervention

Study 3 Inclusion criteria

Population-based sample of community-dwelling adults aged over 18 years old

Exclusion criteria

Individuals with an active episode of major depression, an existing diagnosis of dementia, inability to provide informed consent, impairment of kinetic abilities (ability to use mouse), poor vision, unable to follow verbal commands, diagnosis of aphasia

**Study procedure**

Studies 1, 2, 3 Participants will be recruited via a widespread advertising strategy using flyer dissemination, banners and existing networks.

Study 1 Online MQ Qualtrics surveys will be given to eligible participants to capture demographics, participant’s dementia awareness, mental health and mood, physical activity, cognitive activity, diet, motivation, social networks and quality of life information data (i.e., baseline).

Upon completion of surveys, participants will receive a Brain Bootcamp Box via post containing four physical items representing modifiable risk factors for dementia, an information booklet and their personalised brain health risk profile.

Three months after the Brain Bootcamp box has been sent out, participants will fill out the same assessments (i.e. follow-up)

Study 2 A sample of participants from Study 1 will be invited to participate in a semi-structured interview at follow-up to explore their views of the Brain Bootcamp Box, whether they did/did not implement behaviour change (goal-setting, frequency of item use) and their opinion on the impact of the initiative.

Study 3 Participants from the general public will be recruited as well as a sample of participants from Study 1 will be invited to participate in a traditional and novel memory assessment based on their baseline assessments and dementia risk profile. These participants will engage in a virtual reality cognitive screener developed by Macquarie University, a telephone assessment of their cognition and a feedback questionnaire for their experience with the virtual reality cognitive screener.

**Statistical considerations**

**Sample size calculation**

Study 1 500 older adults are required to detect a meaningful change in dementia risk scores at two time points

Study 2 40 older adults will be suitable to sufficiently address the research question at hand and reach data saturation

Study 3 100 older adults and 100 adults will be recruited to determine sample size for this feasibility study

**Analysis plan**

Study 1 Descriptive analysis of baseline profile of participant demographics (age, gender, economic status) will be presented.

Non-parametric correlations and paired sample t-tests will be conducted to examine and analyse changes in primary outcome (dementia awareness, brain health risk profile) at baseline to three months.

**Study 2** Interview data will be analysed qualitatively for content and themes that emerge, and coded and categorised using N Vivo software

**Study 3** Descriptive statistics will be calculated for participant demographics and domains of the virtual reality screener and the traditional cognitive assessments. This will include percentage of participants who successfully completed the virtual reality cognitive assessments, time taken to complete the virtual reality cognitive assessment module, and scores from the feedback questionnaire.

Correlational analyses and receiver operating curve (ROC) will be conducted for performance scores on the virtual reality assessment module and scores on other cognitive assessment tests.

Duration of the Study 24 months

# Project Team Roles and Responsibilities

**Chief Investigator**

| **Name** | Joyce Siette |
| --- | --- |
| **Title** | Dr |
| **Position Held:** | Research Fellow |
| **Qualifications** | B Psych (Hons), PhD |
| **Role** | As CI, Dr Siette will be responsible for oversight and management, performance, design and finances for all aspects of the research. In addition to these, she will also be leading methods, statistical analysis and write-up of results and reports. |
| **Faculty** | Medicine, Health and Human Sciences |
| **Department** | Australian Institute of Health Innovation |
| **Email address** | [joyce.siette@mq.edu.au](mailto:joyce.siette@mq.edu.au) |

**Co-Investigator**

| **Name** | Christopher Armitage |
| --- | --- |
| **Title** | Professor |
| **Position Held:** | Director of Research, University of Manchester |
| **Qualifications** | BA(Hons) Psych, PhD Psych, PGC(TLHE) |
| **Role** | Assist with study design and write up of results and dissemination |
| **Faculty** | Centre for Health Psychology |
| **Department** | School of Health Sciences |
| **Email address** | [chris.armitage@manchester.ac.uk](mailto:chris.armitage@manchester.ac.uk) |

**Co-Investigator**

| **Name** | Professor Viviana Wuthrich |
| --- | --- |
| **Title** | Professor |
| **Position Held:** | Professor |
| **Qualifications** | M Clin Psych; PhD |
| **Role** | Assist with study design and write up of results and dissemination |
| **Faculty** | Centre for Emotional Health |
| **Department** | Department of Psychology |
| **Email address** | [viviana.wuthrich@mq.edu.au](mailto:viviana.wuthrich@mq.edu.au) |

**Co-Investigator**

| **Name** | Dr Carly Johnco |
| --- | --- |
| **Title** | Dr |
| **Position Held:** | Senior Research Fellow |
| **Qualifications** | M Clin Psych; PhD |
| **Role** | Assist with study design and write up of results and dissemination |
| **Faculty** | Centre for Emotional Health |
| **Department** | Department of Psychology |
| **Email address** | [carly.johnco@mq.edu.au](mailto:carly.johnco@mq.edu.au) |

**Co-Investigator**

| **Name** | Piers Dawes |
| --- | --- |
| **Title** | Associate Professor |
| **Position Held:** | A/Prof |
| **Qualifications** | BSc (Hons); DPhil, |
| **Role** | Assist with study design and write up of results and dissemination |
| **Faculty** | Medicine, Health and Human Sciences |
| **Department** | Linguistics |
| **Email address** | [piers.dawes@mq.edu.au](mailto:piers.dawes@mq.edu.au) |

**Co-Investigator**

| **Name** | Deborah Richards |
| --- | --- |
| **Title** | Professor |
| **Position Held:** | Professor |
| **Qualifications** | BBUS (Comp and MIS0); MAppSc (InfoStudies) |
| **Role** | Assist with study design and write up of results and dissemination |
| **Faculty** | Science and Engineering |
| **Department** | Computing |
| **Email address** | [deborah.richards@mq.edu.au](mailto:deborah.richards@mq.edu.au) |

**Co-Investigator**

| **Name** | Greg Savage |
| --- | --- |
| **Title** | Professor |
| **Position Held:** | Professor |
| **Qualifications** | MSc, Clinical Neuropsych; PhD Psych; BSc(Hons) Psych |
| **Role** | Assist with study design and write up of results and dissemination |
| **Faculty** | Medicine, Health and Human Sciences |
| **Department** | Psychology |
| **Email address** | [greg.savage@mq.edu.au](mailto:greg.savage@mq.edu.au) |

**Co-Investigator**

| **Name** | Paul Strutt |
| --- | --- |
| **Title** | Dr |
| **Position Held:** | Postdoc researcher |
| **Qualifications** | MClinNeuro; PhD Psych |
| **Role** | Assist with study design and write up of results and dissemination |
| **Faculty** | Medicine, Health and Human Sciences |
| **Department** | Cognitive Science |
| **Email address** | [paul.strutt@mq.edu.au](mailto:paul.strutt@mq.edu.au) |

**Co-Investigator**

| **Name** | Kiran Ijaz |
| --- | --- |
| **Title** | Dr |
| **Position Held:** | Research Fellow |
| **Qualifications** | DPhil |
| **Role** | Assist with study design and write up of results and dissemination |
| **Faculty** | Medicine, Health and Human Sciences |
| **Department** | Australian Institute of Health Innovation |
| **Email address** | [kiran.ijaz@mq.edu.au](mailto:kiran.ijaz@mq.edu.au) |

**Co-Investigator**

| **Name** | Kay Deckers |
| --- | --- |
| **Title** | Asst Prof |
| **Position Held:** | Asst Prof |
| **Qualifications** | PhD |
| **Role** | Assist with study design and methodology |
| **Faculty** | School for Mental Health and Neuroscience, Alzheimer Center Limburg |
| **Department** | Maastricht University |
| **Email address** | [kay.deckers@maastrichtuniversity.nl](mailto:kay.deckers@maastrichtuniversity.nl) |

**Co-Investigator**

| **Name** | Irene Heger |
| --- | --- |
| **Title** | Ms |
| **Position Held:** | PhD student |
| **Qualifications** | BS, MS |
| **Role** | Assist with study design and methodology |
| **Faculty** | School for Mental Health and Neuroscience, Alzheimer Center Limburg |
| **Department** | Maastricht University |
| **Email address** | [irene.heger@maastrichtuniversity.nl](mailto:irene.heger@maastrichtuniversity.nl) |

**Co-Investigator**

| **Name** | Sebastian [Köhler](https://www.researchgate.net/profile/Sebastian_Koehler2) |
| --- | --- |
| **Title** | Dr |
| **Position Held:** | Associate Professor |
| **Qualifications** | PhD |
| **Role** | Assist with study design and methodology |
| **Faculty** | School for Mental Health and Neuroscience, Alzheimer Center Limburg |
| **Department** | Maastricht University |
| **Email address** | [s.koehler@maastrichtuniversity.nl](mailto:s.koehler@maastrichtuniversity.nl) |

**Research Assistant**

| **Name** | Laura Dodds |
| --- | --- |
| **Title** | Ms |
| **Position Held:** | Research Assistant |
| **Qualifications** | BA-Psyc, BHlth, MPH |
| **Role** | Assist with recruitment, data collection and write up of results. |
| **Faculty** | Medicine, Health and Human Sciences |
| **Department** | Australian Institute of Health Innovation |
| **Email address** | [laura.dodds@mq.edu.au](mailto:laura.dodds@mq.edu.au) |

**Research Assistant**

| **Name** | Meredith Porte |
| --- | --- |
| **Title** | Ms |
| **Position Held:** | Research Assistant and Programmer |
| **Qualifications** | BIT |
| **Role** | Assist with virtual reality cognition module development |
| **Faculty** | Medicine, Health and Human Sciences |
| **Department** | Psychology |
| **Email address** | [meredith.porte@mq.edu.au](mailto:meredith.porte@mq.edu.au) |

**Co-Investigator**

| **Name** | Professor Johanna Westbrook |
| --- | --- |
| **Title** | Professor |
| **Position Held:** | Professor |
| **Qualifications** | BA, DipArts, MSc, PhD, FCHSM, FACHI, Fsc (Research), RCPA |
| **Role** | Professor Westbrook will provide strategic guidance and advice to the Chief Investigator. |
| **Faculty** | Medicine, Health and Human Sciences |
| **Department** | Australian Institute of Health Innovation |
| **Email address** | [johanna.westbrook@mq.edu.au](mailto:johanna.westbrook@mq.edu.au) |

# 1. BACKGROUND AND INTRODUCTION

### 1.1. Literature Review

Dementia is one of the most common causes of disability, dependency and mortality among older adults and has considerable physical, psychological, social, and economic impacts on individuals diagnosed with dementia, their relatives, formal and informal caregivers, and society at large [1]. Cognitive ageing and dementia are global health priorities, with current estimates suggesting that 50 million people are living with dementia, with that estimate expected to triple by 2050 [2].

Despite extensive global research, there is no curative treatment for dementia [3]. However, there is considerable support for modifiable lifestyle risk factors as contributors to dementia development in later life [4]. Recent estimations suggest that one in three dementia cases may be attributable to common modifiable risk factors [5]. An earlier study similarly concluded that around a third of Alzheimer's disease cases were attributable to modifiable risk factors and estimated that relative reductions of 10% per decade in the prevalence of each of seven key risk factors could reduce Alzheimer's disease prevalence in 2050 by 8.3%[5]. Several healthy living behaviours, such as regular physical exercise, high mental activity and adequate blood pressure control [6], have been recognised as effective risk reduction strategies to delay dementia onset, and are receiving increasing attention in both research and policy [1, 7-10].

Whilst older adults may recognise the link between modifiable risk factors and dementia risk, making and sustaining lifestyle changes is difficult [8]. Innovative initiatives that are less time-intensive, targeting multi-domain lifestyle changes and can be implemented at large-scale to enable individuals to successfully make and sustain changes in their daily routine, is required. One potential approach is to adopt behaviour change principles in public health initiatives. Behaviour change frameworks posit that goal setting and the addition of physical and psychological resources to an individual’s environment can prompt and maintain lifestyle changes [11]. Previously, the most effective interventions to maintain behaviour change in the long-term adopted self-regulation techniques such as goal-setting and progress monitoring strategies [11]. This method was adopted in the Eat Better & Move More (EBMM) program (part of the “Steps to a HealthierUS” public health initiative) which incorporated physical cues (pedometers, tips and task sheets) and information/education resources (guidebooks and mini-talks/videos) to effectively influence behaviour change, with 75% of participants demonstrating improved nutrition and physical activity levels [10]. Thus, creating an educational environment with the provision of mechanisms like physical cues may increase autonomous motivation to adjust habits and patterns [11].

Another important aspect of understanding individual dementia risk is obtaining a cognitive screen. However, there are various barriers to administering and participating in such tests for both clinicians and patients [12-14]. For patients, current health status, societal stigma [15] and pre-existing attitudes, screening pressures, misconceptions and lack of dementia literacy have major implications on seeking a dementia screen [12]. For clinicians, poor prognosis, lack of available treatment options, cost, limited time, and accuracy of the screening tool are significant deterrents to screening operations and follow-ups on positive dementia screens [12]. To address some of these limitations, research into the gamification of cognitive function screeners has seen promising findings. There is evidence to suggest the possibility of memory performance assessed using VR is more reliably associated with general cognitive functioning and may be a more appropriate “age-fair” alternative compared to traditional neuropsychological screens [16]. However, evidence for its acceptance[17], utility and ability to discriminate between age-related cognitive decline and dementia-related cognitive decline across multiple domains is inconsistent and sparse, largely due to studies adopting a cross-sectional design [16, 18].

**Brain Bootcamp**

In response to this backdrop, the *Brain Bootcamp* public health campaign was funded by the New South Wales (NSW) State Government to support healthy and active brain lifestyles and reduce dementia risk in older adults. The delivery of individualised brain health risk score, educational resources and physical items designed to support health-related behaviour change will be packaged in a box and delivered for free to 500 older adults in Sydney, Australia. A sub-set of individuals will also engage in a virtual reality cognitive screener which will help to determine the feasibility of delivering gamified cognitive tests on a larger scale.

In Study 1, our aim is to evaluate the impact of the Brain Bootcamp campaign in reducing dementia risk and improving dementia literacy and knowledge of modifiable lifestyle factors in older Australians. Study 2 aims to understand participants’ views of dementia prevention and explore the acceptability and integration of the intervention into daily life and routine practice. In Study 3, our aim is to determine the feasibility, acceptability and discriminate validity of a virtual reality cognitive screener in a sub-set of older adults.

# 2. HYPOTHESIS

Our hypotheses are that:

1. The Brain Bootcamp campaign will improve older adults’ brain health risk profile at follow-up assessment.
2. The Brain Bootcamp campaign will increase older adults’ dementia awareness and knowledge of modifiable risk factors.
3. The virtual reality cognitive screener is feasible and acceptable for use with younger and older adults.
4. The virtual reality cognitive screener is able to discriminate between cognitively intact and cognitively impaired participants.

# 3. STUDY OBJECTIVES

**Study 1**

### 3.1. STUDY 1 PRIMARY OBJECTIVES

(i) To evaluate the impact of the Brain Bootcamp campaign in increasing dementia awareness and knowledge of modifiable lifestyle factors in older Australians

(ii) To evaluate the impact of the brain bootcamp campaign in improving older adults’ brain health risk profile

**Study 2**

### STUDY 2 PRIMARY OBJECTIVE

To understand participants’ views of dementia prevention and the adoption of Brain Bootcamp into their daily lives.

**Study 3**

### STUDY 3 PRIMARY OBJECTIVES

1. To determine the validity and reliability of new virtual reality cognitive screener in assessing cognitive function in older adults
2. To assess the ability of the virtual reality cognitive screener to discriminate between cognitively intact and cognitively impaired participants.

# 4. Project DESIGN

### 4.1. DESIGN

This project will utilise a mixed-methods approach. Study 1 is a prospective pre post study to evaluate the impact of the multimodal intervention on dementia awareness and risk. Study 2 is a qualitative study designed to assess the experience of participants’ who engaged in the Brain Bootcamp. Study 3 is a feasibility study to determine the acceptability, feasibility and discriminant validity of a new virtual reality technology to assess cognition in older adults.

### 4.2. EXPECTED PARTICIPANT NUMBERS

| Study 1 | Study 2 | Study 3 |
| --- | --- | --- |
| 500 older adults | 40 older adults | 100 older adults and 100 adults |

### 4.3. SETTINGS

| Study 1 | Study 2 | Study 3 |
| --- | --- | --- |
| Data will be collected from older adults living within New South Wales (NSW) | Data will be collected from older adults living in NSW who participated in Study 1 | Data will be collected from older adults and young adults living in NSW |

# 5. STUDY PARTICIPANTS

### 5.1. INCLUSION AND EXLUSION CRITERIA

**Study 1 and Study 2**

**Inclusion**

- Community-dwelling individuals aged 65 years or more on date of consent.
- Access to the internet in order to complete the online assessments.
- Living within NSW.
- Ability to communicate sufficiently in English.

**Exclusion**

- Diagnosis of dementia.
- Diagnosis of severe depression.
- Unable to provide informed written consent.

**Study 3**

**Inclusion**

- Community-dwelling adults aged 18 and over
- Access to the internet in order to complete virtual reality component
- Living in NSW
- Ability to communicate sufficiently in English

**Exclusion**

- Unable to provide informed consent
- Diagnosis of dementia
- Diagnosis of severe depression
- Poor vision
- Unable to follow verbal commands
- Diagnosis of aphasia
- Impairment of kinetic abilities (ability to use mouse)

# 6. STUDY PROCEDURES

6.1. Projected STUDY timeline

The study is predicted to span 24 months. A brief outline is provided in **Table 1**

**Table 1:**

|  | Year 1 | Year 2 |
| --- | --- | --- |
| General admin | --Preparation for ethics application and approval  --Reports: progress | Reports: final  Paper write up and publication |
| Study 1 | --Preparation of Brain Bootcamp box  --Delivery of Brain Bootcamp box  --Participant baseline assessment  --Participant follow-up assessment | --Participant follow-up assessment  --Data entry and cleaning  --Statistical analysis |
| Study 2 | --Data collection for process evaluation | --Data entry and cleaning  --Statistical analysis |
| Study 3 | --Virtual reality design and development  --Recruitment of participants  --Administration of traditional and novel assessments  --Feedback survey completion | --Administration of participant and novel assessments  --Feedback survey completion  --Data entry and cleaning  --Statistical analysis |

### 6.2. STUDY PLAN

**Overview**

**Study 1**

This project aims to explore the effectiveness of Brain Bootcamp in improving dementia awareness and knowledge of dementia risk factors amongst the community.

Participants will be adults aged 65 years and older and meet the inclusion criteria (See Section 5.1.)

We will recruit community-dwelling older adults from the general population of NSW through a widespread advertising strategy (**see 6.3**) to invite them to register for Brain Bootcamp and participate in our online Qualtrics surveys. They will be asked to complete our dementia awareness survey, motivation, and complete information on their basic demographics (e.g. age, gender, education, income) as well as information pertaining to brain health risk profile (**see Section 7**). Upon completion they will receive a Brain Bootcamp Box with four physical items and their personalised brain health risk profile based on the LIBRA index[19] to encourage them to adopt healthier habits and behaviours.

Three months after completing the baseline surveys, participants will be asked to complete a follow-up survey which will be the same as the baseline surveys to determine if there has been any change. Participants will receive their updated brain health risk profile.

**Study 2**

A sample from Study 1’s participants will be invited for semi-structured interviews at the end of the follow-up assessments. Interviews will explore their views of the information they received from their profile, whether they have accessed the resources in their Brain Bootcamp pack, any health behaviour change goals, how they did/did not incorporate recommendations into their daily life, and their perception of the overall impact of the initiative. We will also investigate why participants did not complete the trial by including a sample of those who dropped out.

An opt-in approach to recruitment of participants has been selected, to reduce any perceived pressure, and ensure that clients’ decision to participate is made freely. Participants will receive the following information prior to choosing to take part in the study:

- There will be no consequence or penalty associated with choosing not to participate, including any kind of consequence regarding the Brain Bootcamp box previously sent to them.
- Their responses will remain entirely anonymous, and no individual participant will be identified when reporting results from the interviews and focus groups, or any other data analyses.

**Study 3**

General public participants who meet the inclusion criteria and are over the age of 18 will be recruited to participate in both a virtual reality cognitive screener and a traditional telephone based cognitive screening test. For the older adults, a sub-set of older adult participants from Study 1 will be contacted via email to see if they are interested in participating. This will be based on their scores in the brain health risk profile, identified using the LIBRA index, which is shown to be a valid predictor of the development of dementia [19-21]. Younger adults will be recruited from the general public via social media and other relevant channels. All participants will be provided with an opportunity to participate in a feedback survey after they have completed the virtual reality screener. Questions will be about the acceptance, perception and experience of virtual reality.

### 6.3. PARTICIPANT RECRUITMENT AND SCREENING

**Study 1 and Study 2**

Participants will be consecutively recruited through a widespread advertising strategy, including standardised public advertisements and flyers disseminated at general medical practices, community newsletters, local print media, flyers and radio in NSW (**Figure 1**). The general population who are interested in receiving a Brain Bootcamp Box will register online (<http://www.brainbootcamp.com.au>) between January and March 2021. Upon registration they will be asked questions pertaining to the eligibility criteria and eligible members will be prompted to proceed and participate in the campaign. Eligible participants will be asked to provide informed consent on the online registration form. Research assistants will keep a screening log of potentially eligible participants and reasons for exclusion or possible refusal will be recorded.

**Figure 1. Advertising strategy**


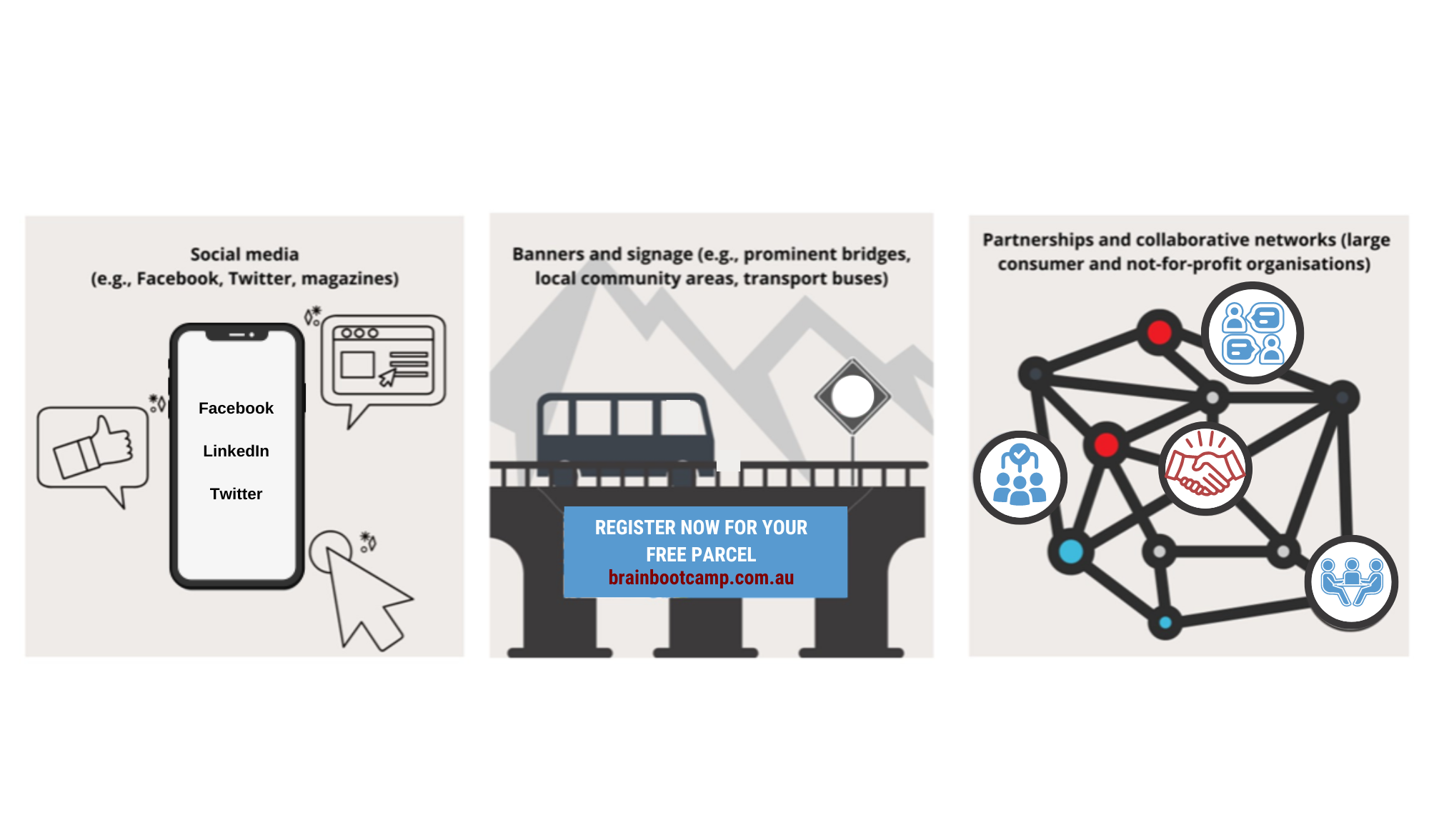


**Study 3**

General public participants older adult and younger adult participants will be recruited via (i) study 1 or (ii) advertisements circulated via social media and researcher contacts. Researchers will determine their eligibility by checking them against the eligibility criteria.

### 6.4. PARTICIPANT ENROLMENT

**Study 1**

The participant will be enrolled into the study after the informed consent process has been completed. We request general public participants to include identifiable information on the surveys if they wish to complete the surveys at two time points. Upon completion of the first survey, participants will receive a study enrolment number which will be documented on all study documents and pre-populated on their second survey to enable the research team to link their results.

**Study 2**

Participants from study 1 will be contacted by researchers to participate in study 2 in which they will provide online consent and be enrolled into the study once this has been provided. Potential participants for the interviews will be assessed against the inclusion and exclusion criteria. If they meet all criteria they will then be enrolled in the study once informed consent is received

**Study 3**

The participant will be enrolled into the study after the informed consent process has been completed. We request general public participants to include identifiable information to ensure they can be contacted to participate in both the traditional cognitive assessment and virtual reality cognitive screener within the same week. Upon completion of the first screening assessment general public participants will receive a study enrolment number which will be pre-populated on the second screening assessment to enable the research team to link their results. Study 1 participants who wish to participate in study 3 will continue to use their study 1 enrolment number. After completing the virtual reality screener participants will be invited to participate in a short online feedback survey.

### 6.5. INFORMATION AND CONSENT

***Overview***

**Study 1**

Study 1 participants are consenting to (1) participate in an online surveys; (2) provide identifiable information at the first administration of surveys; (3) complete the same surveys 3 months later

Participants will visit our website (<http://makemywebdesign.com/brainbootcamp/>) and be able to register their interest in receiving the Brain Bootcamp box. Participants will be sent an automated email with a link to participate in the online survey. At this point, participants will indicate that they fulfill the inclusion criteria (i.e. no diagnosis of dementia, are aged 65 and over, are living within NSW, no diagnosis of severe depression). Participants who meet the eligibility criteria and wish to complete the survey will provide online consent before undertaking the assessments by ticking the consent box.

**Study 2**

Study 2 participants are consenting to (1) participate in a telephone interview to provide feedback on the campaign. At time of follow-up assessment, participants will be contacted and provided with an opportunity to provide their opinion on the Brain Bootcamp campaign. They will be asked to provide consent to participate in a telephone interview that will be recorded and transcribed. We will attempt to reach out to individuals who dropped out of the study (i.e. those who did not update their brain health profile at three-month follow-up).

**Study 3**

Study 3 will include community-dwelling participants aged 18 years and over. Study 3 participants are consenting to participate in a (1) using identifiable information provided in study 1 to be contacted for study 2; (2) participate in a virtual reality cognitive screener or; (3) participate in a traditional cognitive assessment via telephone (4) participate in a feedback survey about the acceptability and feasibility of the Virtual Reality Cognitive Screener .

Participants from Study 1 will be given the opportunity to participate in Study 3 based on their brain health profile at baseline. Adults from the general public participants not involved in the Brain Boot Camp campaign will also be recruited to participate in the study. For the virtual reality cognitive screener, participants will provide online consent and receive a link to participate in the virtual reality screener online. For the traditional cognitive assessment via telephone, researchers will also seek verbal consent at time of administration before undertaking the assessment to ensure participants understand the study. After completion of the virtual reality screener participants will be invited to participate in a brief online feedback survey.

***Consent***

**Study 1 and Study 2**

This study requires the general public aged over 65 to undertake an online surveys assessing their dementia awareness, motivation, alcohol consumption, smoking habits, general mental health and mood, quality of life, cognitive and physical activity levels, and an inventory of their social network. Participants will be required to provide identifiable information to be contacted to complete the same online surveys, 3 months later. Therefore, participants are consenting (1) participate in online surveys; (2) provide identifiable information at the first administration of the surveys and (3) complete the same surveys 3 months later; (4) contacted for a telephone interview to provide feedback on the campaign.

**Study 3**

This study requires the general public aged over 65 to undertake a new virtual reality cognitive screener (online) or a telephone-based cognitive assessment. Participants will be required to provide consent for researchers to use the identifiable information provided in study 1 to be contacted to participate in study 2. Therefore, participants are consenting to (1) using identifiable information provided in study 1 to be contacted for study 2; (2) participate in a virtual reality cognitive screener or; (3) participate in a traditional cognitive assessment via telephone (4) participate in an online feedback survey about the acceptability and feasibility of the Virtual Reality Cognitive Screener

Most research involving this population is premised upon the informed consent of the participants. If older participants are not competent enough to make a decision due to cognitive impairments, they will be excluded from the study. This is highlighted in the inclusion criteria.

Participant capacity to consent will be made explicit by them completing the survey and providing the research team with their online consent.

Information sheets and consent forms will provide an explicit outline of the study, participant involvement and expectations with a provision for “opt out” and Project Manager contact details for any queries. These documents will clearly state that the participants may opt out at any time.

### 6.6. DATA COLLECTION

**Study 1**

A widespread advertising strategy (**see Section 6.3**) will be used to recruit participants via the Brain Bootcamp website. Information about basic demographics (name, age, sex, phone number, address, marital status, education), dementia awareness, motivation, alcohol consumption, smoking habits, general mental health and mood, quality of life, cognitive and physical activity levels, and an inventory of their social network will be collected as described in **Section 7**. The surveys will take up to 25 minutes in total to complete. Participants will be provided with their individualised results in their Brain Bootcamp box alongside contact details of researchers if they wish to discuss their results.

**Study 2**

Participants who wish to participate in a telephone interview will share information on their views of the information they received from their profile, whether they have accessed the resources in their Brain Bootcamp box, any health behaviour change goals, how they did/did not incorporate recommendations into their daily life, and their perception of the overall impact of the campaign. Interviews will be audio recorded and will be sent of a to a professional transcribing service to be transcribed verbatim then the audio-recording destroyed. Typed transcripts will be edited to remove any identifying information. Each interview should take approximately 15 minutes.

**Study 3**

Participants will be recruited from the general public who are over 18. Study 1 participants will be contacted to give them an opportunity to participate in Study 3, based on their brain health risk scores. Information about their memory performance will be collected as outlined in **Section 7.** Both the Virtual Reality Cognitive Screener and telephone based cognitive assessment will take approximately 30 minutes to complete. A random selection of half of each of the younger and older adults participants will receive the traditional telephone cognitive assessment (Group A), whilst the other half will complete the Virtual Reality Cognitive Screener (Group B). Then, the groups will swap and Group A will complete the Virtual Reality Cognitive Screener , whilst Group B completes the telephone cognitive assessment. Both assessments will then be complete within a week of each other. After completion of the virtual reality cognitive screener, participants will be invited to provide feedback via a voluntary online survey about the feasibility and acceptability of their virtual reality experience.

### 6.7. END OF STUDY WITHDRAWAL PROCEDURE

Participants will receive a summary written specifically for their understanding of the aggregate results if they wish.

### 6.8. PATIENT WITHDRAWAL

Participants will be able to withdraw at any point of the study if they no longer want to be involved. Participants can withdraw by informing the researchers.

# 7. OUTCOME MEASURES

Participants will have the following measures assessed at two time points (baseline and three months later). A summary of measures used in the current study are provided in **Table 2**, with further detail below.

**Table 2. Assessment measures for participants**

| Study 1 | | |
| --- | --- | --- |
| Outcome | Data collection instrument | |
| Primary Outcomes | | |
| Dementia Awareness | Dementia Awareness Questionnaire [22, 23] | |
| Brain health risk profile | LIBRA index [19] | |
| Motivation | Motivation to Change Lifestyle and Health Behaviour for Dementia Risk Reduction (MCLHB-DRR)[24] | |
| Quality of life | EuroQol Group EQ-5D-5L [25] | |
| Social networks | Lubben Social Network Scale (LSNS-6) [26] | |
| Overall experience | Impact Evaluation survey [27] | |
|  |  |  |
| Study 2 | | |
| Outcomes | | |
| Behaviour change/ impact of campaign | Semi-structured interviews | |
| Study 3 | | |
| Outcomes | | |
| Memory performance | Virtual reality assessment (developed in house by our researchers)  Telephone Interview for Cognitive Status-Modified (TICS-M) | |
| Acceptability | Research-team generated feedback survey | |

**Study 1**

**Dementia awareness:** Dementia awareness and literacy will be measured using the Dementia Awareness Questionnaire [23]. This questionnaire includes ten items from the UK’s BSA survey [22], assessing self-reported knowledge of dementia, personal experience with people with dementia, dementia risk awareness and knowledge of four modifiable dementia risk and protective factors (i.e., hypertension, smoking, physical activity, depression and diabetes). We will further include nine additional modifiable risk and protective factors (i.e., obesity, coronary heart disease, chronic kidney disease, hypocholesteraemia, mental activity, low to moderate alcohol intake, healthy diet, midlife hearing loss and social connections), in order to assess all the known modifiable risk and protective factors in the literature [28, 29]. In addition, three sham factors will be included (use of painkillers, personal hygiene and having children) to check for monotone answering tendency. Additional items will be developed to evaluate the needs, wishes and barriers of participants concerning brain health, such as the need for further information, preferred information source, subjective barriers to engage in a brain-healthy lifestyle, and motivation to use an internet application to increase risk factor awareness. Most items will be set up as statements. Participants will be asked to what extent they agree or disagree on a 5-point Likert scale ranging from ‘strongly disagree’ to ‘strongly agree’. Improvements in dementia literacy are defined as the presence of one to three standard deviations improvement on the questionnaire compared to baseline.

**Demographics** including age, gender, income, marital status, address, , education level (as identified by completed years of schooling and tertiary studies)

**Depression:** Participants will self-report depressive symptoms using the 9-item brief depression severity measure, Patient Health Questionnaire (PHQ-9). Responses range from 0 (“not at all”) to 3 (“nearly every day”) [30]. It has been used in several contexts including medical settings and amongst the general population [31] and has a sensitivity and specificity of 88% for major depression [30]. A score 10 or above indicates symptoms of minor depression, dysthymia or major depression (mild)

**Physical activity:** Levels of physical activity will be measured based on the short LIBRA administration used in the *MijnBreincoach app* developed for online administration*.* It consists of one question with three possible responses “Yes” (0), “A little” (0) and “No” [23].

**Social networks:** will be measured using the 6-item version of the Lubben Social Network Scale (LSNS-6). Internal reliability (0.83) is good and the two sub-scales (family and friendships) both demonstrate high levels of internal consistency. Scores range from 0-30 with higher scores indicating more social engagement and scores < 12 identifying individuals at risk of social isolation[26].

**Quality of life:** will be measured using the EQ-5D-5L scale. It consists of a self-administered health index and a 100-point visual analogue scale (VAS), for participants to rate their current health state from 0 as their ‘worst imaginable health state’ to 100, their ‘best imaginable health state’ [25]. The instrument covers mobility, self-care, pain/discomfort, usual activities and anxiety/depression[32]. Utility scores are quantified along a continuum that ranged from -0.59 (worst health) to 1.00 (perfect health). It has demonstrated convergent validity [33] and good reliability for individuals with diverse health conditions [34, 35].

**Cognitive activity:** Will be measured using Adapted Cognitive Reserve questionnaire (CRIq)[36]. This 20 item instrument measures cognitive activity across three sections; education (e.g. years of education, training courses), working activity (e.g. adulthood professions) and leisure time (e.g. reading newspaper, playing music, social and physical activities)[36]. Performance below the cut-off in at least 2 sections indicates a lower risk of cognitive impairment [37] This instrument has established construct validity in unhealthy populations.

**Diet:** adherence will be measured using the Mediterranean Diet Adherence Screener (MEDAS)[38]. This is a validated[39] a 14-item scale and a score of 0 or 1 is assigned to each item. A maximum score of 14 indicates the greatest adherence to the diet.

**Motivation:** Motivational attitudes and beliefs to modify lifestyle specific to dementia risk reduction will be measured using Motivation to Change Lifestyle and Health Behaviour for Dementia Risk Reduction (MCLHB-DRR)[24]. This is a 27-item tool, commonly used in the evaluation of dementia prevention programs. It has been validated amongst individuals aged 50 and over with moderate to high internal reliability and test-retest reliability.

**Other factors**

- Heart disease (heart or blood vessel condition; yes, no, I don’t know);
- Low to moderate alcohol consumption (standard units consumed per day);
- Smoking habits (i.e. current smoker; yes or no);
- Chronic kidney disease (yes, no, I don’t know)
- Cholesterol (High cholesterol; yes, no, I don’t know)

**Impact Evaluation**

The evaluation will be based on these overarching questions from the Australian Government impact evaluation framework [27]:

- Did the initiative make a difference?
- How much of a difference did the initiative make?
- For whom, in what situations, and in what ways did the initiative make a difference?
- To what extent can a specific impact be attributed to the initiative?
- How did the initiative make a difference?
- Will the initiative work elsewhere?
- What is needed for the initiative to work elsewhere?

**Study 2**

A semi-structured questioning route beginning with general opening questions and several key questions and sub questions. The questioning route will be organised into themes; (1) Brain Bootcamp overall; (2) Effectiveness of intervention strategies (a) brain health risk profile (b) Brain Bootcamp box items (3) Recommendations and Improvements. Questions have been developed based on feasibility and acceptance interviews employed in previous public health interventions[40-42].

**Study 3**

Participants will be divided into two groups (Group A and Group B) and administered the Virtual Reality Cognitive Screener (TICS-M) and the Telephone Interview for Cognitive Status-Modified within one week.

A flow chart of Study 3 is provided below:


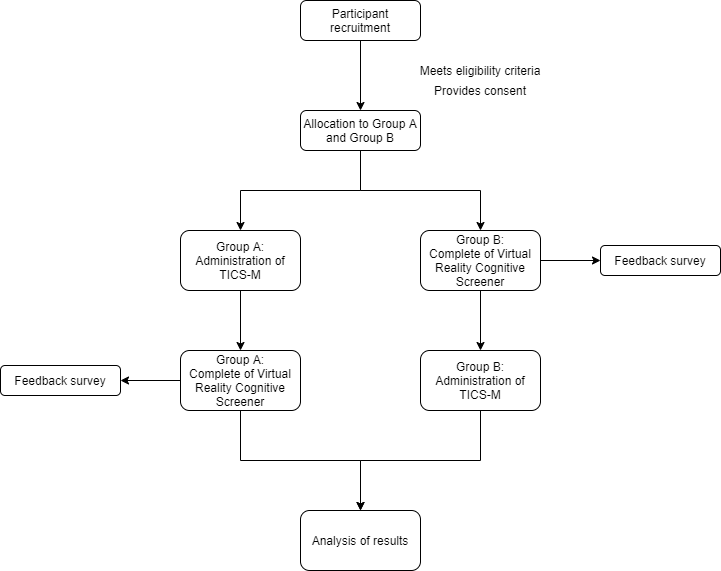


**Memory Performance:**

Traditional: Classification of cognitive status will be conducted by administering a 23 question Telephone Interview for Cognitive Status-Modified (TICS-M) either in person on over the phone. This will include scoring on name, date, age, phone number, ability to count backwards from 20 to 1, immediate recall of 10 words, counting backwards from 100 by 7, the naming of objects and concepts, repetition of phrases, the name of current political figures, tapping 5 times, naming opposites of common words, and delayed recall of 10 words. The maximum score is 50 points. The TICS-M will classify individuals as having no or minimal cognitive impairment (>40) or possible mild cognitive impairment (≤ 20) or moderate cognitive impairment (≤12). The TICS-M has been previously validated [43] with strong psychometric properties[44]. Prior studies have shown that the TICS-M has a high sensitivity in the detection of dementia[45, 46] but a low positive predictive value [43].

A further 5 minutes will be used to calculate the scores and to disseminate this finding to the participant at the end of the session. Whilst researchers are happy to discuss the findings further if the client wishes, if any participant has been identified as having a low memory score <27 they will be referred to their local GP to obtain a more comprehensive assessment.

Virtual Reality Cognitive Screener (VRCS): Developed using the Unity game engine, the application places the user on a virtual in a virtual restaurant modelled to be similar to an Australian café. The user’s role will be that of the waiter, needing to serve restaurant customers. The VRCS will be developed using Unity’s WebGL build option. This will allow the VRCS to run in current versions of most major web-browsers. Suggested browsers are (in no particular order) Microsoft Edge, Mozilla Firefox and Google Chrome. (Videos of the VRCS in development are uploaded in “Other Project Material”)

Using the VRCS, participants will gain a score out of 165. The score will be achieved by the participant serving up to five tables of customers correctly – at the most basic level this consists of one table of two customers and at the highest level this will be four tables of four customers. Participants are asked to remember the items ordered by customers from a particular table and to correctly collect the food items from the kitchen and bring it back to the right customer. The complexity of a customer orders will increase with each table.

The VRCS is designed to assess four cognition domains which is briefly described below:

1. Free recall: To correctly input the customer’s order onto a virtual whiteboard immediately after taking the order
2. Delayed recall: To correctly re-input the customer’s order onto the virtual whiteboard after a typical distractor task (e.g., customer rings to book a table)
3. Cued recall: To correctly identify the customer’s order on the kitchen counter amongst other food orders
4. Spatial recall: To bring the customer order back to the right table to the right customer.

As this is still in the development and is not a validated measure to reliably predict memory scores, participants will not be informed of their score. They will only be provided with the number of orders they got correct at the end of the game.

**Feedback survey:** Participants will also be invited to participate in an online feedback survey once the Virtual Reality Cognitive Screener is completed. The survey will be about the acceptability and feasibility of the Virtual Reality Cognitive Screener. Questions have been developed based on previous evaluation of virtual reality screeners used in health and medical settings with older adults [42]. There are six questions, and the answers will be rated on a 5-point Likert scale (1 - strongly disagree, 2 - disagree, 3 - neutral, 4 - agree, 5 - strongly agree). Total feedback scores will be calculated by summing the scores from the six questions.

# 8. STATISTICAL CONSIDERATIONS

### 8.1. SAMPLE SIZE OR POWER CALCULATION

**Study 1**

A sample size of 403 participants will be needed to detect a meaningful change in dementia risk scores (-0.3 Z score, SD = 0.5) between baseline and three-month follow-up (with 90% power and α=0.05), and a drop out rate of 25%.

**Study 2**

A theoretical sampling approach will be used to select participants based on their analytical relevance to the study question, in order to achieve the experience of individuals with different dementia risk profiles and those who dropped out of the study. Sampling will end once saturation has been reached (i.e., no new themes are emerging, and the research findings are coherent and explicable). We estimate that this will involve a sample size of 40 participants.

**Study 2**

Based on a power analysis for biserial correlation using G*Power 3 with a conservative medium effect of ρ = .30 [47]report very high effect sizes with a similar design), and 80% power, an N of 64 is required to detect an association at p = .05 for each group. The final proposed sample size (N=100 per group) was larger than required for the present study to account for potential drop-outs between assessments.

### 8.2. PROVIDE A DETAILED ANALYSIS PLAN

**Study 1**

All surveys will be visually inspected for completeness on collection to minimise missing data. Assessment data will be entered into databases and analysed using standard statistical software (eg. SPSS V.22). Quantitative data obtained in response to structured assessment items will be entered, cleaned, analysed and summarised. The investigators will use descriptive statistics to describe the study group and to summarise their use of various components of the Brain Bootcamp Pack over the course of three months. Paired sample t tests will be used to analyse changes in our primary outcome, brain health risk profile, from baseline to three months. Nonparametric correlations will be conducted to examine the relationship between secondary outcomes (social networks, quality of life, motivation and dementia awareness) and changes in primary outcome.

**Study 2**

Interview data that are obtained in the semi-structured interviews will be analysed qualitatively for content and themes that emerge, and coded and categorised using N Vivo software. Qualitative data analysis will involve an initial open coding of all transcriptions, followed by axial coding using grounded theory techniques whereby initial codes, indicators and concepts are triangulated with other findings (e.g., observational, and quantitative) leading to refined analytical levels, relevant to the study aims.

**Study 3**

Descriptive data will be compared between the groups using a two-sample t test and the Fisher exact test for continuous and categorical variables, respectively. Summary statistics will be calculated individually for the Virtual Reality Cognitive Screener, traditional cognitive assessment and questionnaire-dependent feedback scores. Younger and older adults (and older adults with cognitive impairment) cognitive performance on all measures will be compared using the two-sample t tests.

Performance scores will be analysed at each task level, given a total score, and compared between the older cognitive-intact and younger groups using the methods described above, as appropriate. Correlation between performance scores and other variables will assessed using Pearson correlation. A logistic regression will performed based on the total performance scores, followed by ROC analysis to assess its predictive capability to discriminate between cognitively intact and cognitively impaired individuals. Analyses will be performed using SPSS V22.0.

# 9. QUALITY CONTROL AND ASSURANCE

### 9.1. CONTROL OF DATA CONSISTENCY

Data analysis will be undertaken using appropriate statistical programming software (e.g. SPSS) and user-written code will be saved in a shared location to enable replication and version control. Transcribed interviews will be redacted and identifiable information removed before qualitative analysis and coding. We will conduct appropriate data cleaning, harmonisation, standardisation and internal consistency checks.

### 9.2. AUDITS

A Project Management Committee will be established with the lead researchers. It will meet monthly and be based on the strong communication networks established from prior research projects.

### 9.3. PROTOCOL AMENDMENTS

Any amendments to the protocol will be reported to the Macquarie University Human Ethics Committee during the study.

# 10. ETHICS

### 10.1. INVESTIGATOR AUTHORISATION PROCEDURE

Ethical approval from the University of Macquarie (Medical Sciences) HREC will be gained prior to commencement of participant recruitment.

### 10.2. PATIENT PROTECTION

The responsible researchers will ensure that the study is completed in accordance with the guidelines set out in the [*National Statement on Ethical Conduct in Human Research*](http://www.nhmrc.gov.au/guidelines/publications/e72) (2007) (the *National Statement*) and the [*CPMP/ICH Note for Guidance on Good Clinical Practice*](http://www.tga.gov.au/industry/clinical-trials-note-ich13595.htm) and any other relevant legislation/guidelines.

# 11. CONFIDENTIALITY AND STORAGE AND ARCHIVING OF STUDY

Data collected in Studies 1 and 3 will be re-identifiable and stored electronically on a password-protected server. Only researchers approved by the Macquarie University Human Research Ethics Committee will have access to these data. Records will be retained for 5 years from the date of the most recent publication in accordance with the Records Act ‐ General Retention and Disposal Authority University Records (GDA 23) (2005) and the Australian Code for the Responsible Conduct of Research (2007).

All Qualtrics survey responses will be *stored on secure servers in California and only* researchers approved by the Macquarie University Human Research Ethics Committee will have access to these data.

Following completion of the interview in study 2, the audio-recording will be transcribed and then the audio-file destroyed. Typed transcripts will be edited to remove any identifying information. Only researchers from Macquarie University and the professional transcriber will have access to these data. The professional transcribing service will maintain confidentiality as per the ethics application.

Any data presented in publications, conferences or reports will be non-identifiable.

# 12. RESULTS, OUTCOMES AND FUTURE PLANS

Results

Results will be disseminated through the usual research outputs such as conference paper(s) and peer-reviewed publications, as well as through information updated on the Australian Institute of Health Innovation website.

Outcomes

The study will provide an in-depth insight into how public health campaigns targeting improved literacy on dementia risk and protective factors can be successfully conducted. Our campaign, which consists of education, goal setting and use of physical cues to maintain risk reduction behaviors will empower participants to implement personal changes into everyday life.

Using a multidimensional perspective, this study will provide a detailed understanding of the importance of social and functional factors for either the decline or maintenance of cognition. It will further assist in targeting both individuals at high risk of dementia development and those who are most likely to benefit from protective factors, therefore guiding optimal clinical care and follow-up procedures. Further, this study has the potential to re-orient the future of health services within an evolving digital context through the development and trial of a virtual reality memory assessment which may have benefits for health professionals, individuals and the community in the long-term.

Future Plans

Additional funding will be sought to expand the study’s design (e.g., approach these questions using a randomised controlled design).

# 13. TRIAL SPONSORSHIP AND FINANCING

The study is funded by NSW Government “My Community Project” Grant.

# 14. REFERENCES

1. Livingston, G., et al., *Dementia prevention, intervention, and care.* Lancet, 2017. **390**(10113): p. 2673-2734.

2. Alzheimer's Research UK. *Global Prevalence*. 2018; Available from: <https://www.dementiastatistics.org/statistics/global-prevalence/>.

3. Rogers, N.T., A. Steptoe, and D. Cadar, *Frailty is an independent predictor of incident dementia: Evidence from the English Longitudinal Study of Ageing.* Sci Rep, 2017. **7**(1): p. 15746.

4. O'Donnell, C.A., et al., *Promoting modifiable risk factors for dementia: is there a role for general practice?* Br J Gen Pract, 2015. **65**(640): p. 567-8.

5. Norton, S., et al., *Potential for primary prevention of Alzheimer's disease: an analysis of population-based data.* Lancet Neurol, 2014. **13**(8): p. 788-94.

6. Downey, A., et al., *Preventing Cognitive Decline and Dementia: A Way Forward.* 2017: National Academies Press.

7. Anstey, K.J., et al., *Joining forces to prevent dementia: The International Research Network On Dementia Prevention (IRNDP).* Int Psychogeriatr, 2017. **29**(11): p. 1757-1760.

8. Fratiglioni, L. and C. Qiu, *Prevention of cognitive decline in ageing: dementia as the target, delayed onset as the goal.* Lancet Neurol, 2011. **10**(9): p. 778-9.

9. Gorelick, P.B., et al., *Defining Optimal Brain Health in Adults: A Presidential Advisory From the American Heart Association/American Stroke Association.* Stroke, 2017. **48**(10): p. e284-e303.

10. Lincoln, P., et al., *The Blackfriars Consensus on brain health and dementia.* Lancet, 2014. **383**(9931): p. 1805-6.

11. Samdal, G.B., et al., *Effective behaviour change techniques for physical activity and healthy eating in overweight and obese adults; systematic review and meta-regression analyses.* International Journal of Behavioral Nutrition and Physical Activity, 2017. **14**(1): p. 42.

12. Martin, S., et al., *Attitudes and preferences towards screening for dementia: a systematic review of the literature.* BMC Geriatrics, 2015. **15**(1): p. 66.

13. Boustani, M., et al., *Measuring primary care patients' attitudes about dementia screening.* International journal of geriatric psychiatry, 2008. **23**(8): p. 812-820.

14. Phillips, J., D. Pond, and S. Goode, *Timely diagnosis of dementia: can we do better.* Canberra: Alzheimer’s Australia, 2011.

15. Krohne, K., Å. Slettebø, and A. Bergland, *Cognitive screening tests as experienced by older hospitalised patients: a qualitative study.* Scandinavian journal of caring sciences, 2011. **25**(4): p. 679-687.

16. La Corte, V., et al., *Episodic Memory Assessment and Remediation in Normal and Pathological Aging Using Virtual Reality: A Mini Review.* Frontiers in Psychology, 2019. **10**(173).

17. Robillard, J.M., et al., *Patient perspectives of the experience of a computerized cognitive assessment in a clinical setting.* Alzheimer's & dementia (New York, N. Y.), 2018. **4**: p. 297-303.

18. Parsons, T.D. and M. Barnett, *Validity of a Newly Developed Measure of Memory: Feasibility Study of the Virtual Environment Grocery Store.* J Alzheimers Dis, 2017. **59**(4): p. 1227-1235.

19. Schiepers, O.J.G., et al., *Lifestyle for Brain Health (LIBRA): a new model for dementia prevention.* Int J Geriatr Psychiatry, 2018. **33**(1): p. 167-175.

20. O’Donnell, C.A., et al., *Reducing dementia risk by targeting modifiable risk factors in mid-life: study protocol for the Innovative Midlife Intervention for Dementia Deterrence (In-MINDD) randomised controlled feasibility trial.* Pilot and Feasibility Studies, 2015. **1**(1): p. 40.

21. Deckers, K., et al., *Target risk factors for dementia prevention: a systematic review and Delphi consensus study on the evidence from observational studies*. 2015. p. 234-246.

22. Marcinkiewicz A and Reid S, *Attitudes to dementia: Findings from the 2015 British Social Attitudes survey,*. 2016: London.

23. Heger, I., et al., *Dementia awareness and risk perception in middle-aged and older individuals: baseline results of the MijnBreincoach survey on the association between lifestyle and brain health.* BMC Public Health, 2019. **19**(1): p. 678.

24. Sarang, K., et al., *Development of the Motivation to Change Lifestyle and Health Behaviours for Dementia Risk Reduction Scale.* Dementia and geriatric cognitive disorders extra, 2014. **4**(2): p. 172-183.

25. EuroQol Group, *EuroQol-a new facility for the measurement of health-related quality of life.* Health Policy, 1990. **16**(3): p. 199-208.

26. Lubben, J., et al., *Performance of an Abbreviated Version of the Lubben Social Network Scale Among Three European Community-Dwelling Older Adult Populations.* The Gerontologist, 2006. **46**(4): p. 503-513.

27. Rogers, P., et al., *Choosing appropriate designs and methods for impact evaluation.* Office of the Chief Economist, Department of Industry, Innovation and Science, Australian Government, 2015.

28. Vos, S.J.B., et al., *Modifiable Risk Factors for Prevention of Dementia in Midlife, Late Life and the Oldest-Old: Validation of the LIBRA Index.* J Alzheimers Dis, 2017. **58**(2): p. 537-547.

29. Deckers, K., et al., *Lack of associations between modifiable risk factors and dementia in the very old: findings from the Cambridge City over-75s cohort study.* Aging Ment Health, 2018. **22**(10): p. 1272-1278.

30. Kroenke, K., R.L. Spitzer, and J.B. Williams, *The PHQ-9: validity of a brief depression severity measure.* J Gen Intern Med, 2001. **16**(9): p. 606-13.

31. Martin, A., et al., *Validity of the Brief Patient Health Questionnaire Mood Scale (PHQ-9) in the general population.* General Hospital Psychiatry, 2006. **28**(1): p. 71-77.

32. Lawton, M.P., *Assessing quality of life in Alzheimer disease research.* Alzheimer Dis Assoc Disord, 1997. **11 Suppl 6**: p. 91-9.

33. Janssen, M.F., et al., *Measurement properties of the EQ-5D-5L compared to the EQ-5D-3L across eight patient groups: a multi-country study.* Quality of life research : an international journal of quality of life aspects of treatment, care and rehabilitation, 2013. **22**(7): p. 1717-1727.

34. Hernandez, G., et al., *EuroQol (EQ-5D-5L) Validity in Assessing the Quality of Life in Adults With Asthma: Cross-Sectional Study.* Journal of medical Internet research, 2019. **21**(1): p. e10178-e10178.

35. Bilbao, A., et al., *Psychometric properties of the EQ-5D-5L in patients with hip or knee osteoarthritis: reliability, validity and responsiveness.* Qual Life Res, 2018. **27**(11): p. 2897-2908.

36. Nucci, M., D. Mapelli, and S. Mondini, *Cognitive Reserve Index questionnaire (CRIq): A new instrument for measuring cognitive reserve.* Aging clinical and experimental research, 2011. **24**.

37. Kartschmit, N., et al., *Measuring Cognitive Reserve (CR) - A systematic review of measurement properties of CR questionnaires for the adult population.* PloS one, 2019. **14**(8): p. e0219851-e0219851.

38. Martínez-González, M.A., et al., *A 14-item Mediterranean diet assessment tool and obesity indexes among high-risk subjects: the PREDIMED trial.* PloS one, 2012. **7**(8): p. e43134-e43134.

39. Schröder, H., et al., *A Short Screener Is Valid for Assessing Mediterranean Diet Adherence among Older Spanish Men and Women.* The Journal of Nutrition, 2011. **141**(6): p. 1140-1145.

40. De Cocker, K., et al., *Acceptability and feasibility of potential intervention strategies for influencing sedentary time at work: focus group interviews in executives and employees.* International Journal of Behavioral Nutrition and Physical Activity, 2015. **12**(1): p. 22.

41. Northridge, M.E., et al., *A Protocol for a Feasibility and Acceptability Study of a Participatory, Multi-Level, Dynamic Intervention in Urban Outreach Centers to Improve the Oral Health of Low-Income Chinese Americans.* Frontiers in Public Health, 2018. **6**(29).

42. Chua, S.I.L., et al., *Virtual Reality for Screening of Cognitive Function in Older Persons: Comparative Study.* J Med Internet Res, 2019. **21**(8): p. e14821.

43. Welsh, K.A., J.C. Breitner, and K.M. Magruder-Habib, *Detection of dementia in the elderly using telephone screening of cognitive status.* Neuropsychiatry, Neuropsychology, & Behavioral Neurology, 1993. **6**(2): p. 103-110.

44. Buckwalter, J.G., V.C. Crooks, and D.B. Petitti, *A Preliminary Psychometric Analysis of a Computer-Assisted Administration of the Telephone Interview of Cognitive Status-Modified.* Journal of Clinical and Experimental Neuropsychology, 2002. **24**(2): p. 168-175.

45. Gallo, J.J. and J.C. Breitner, *Alzheimer's disease in the NAS-NRC Registry of aging twin veterans, IV. Performance characteristics of a two-stage telephone screening procedure for Alzheimer's dementia.* Psychol Med, 1995. **25**(6): p. 1211-9.

46. Beeri, M.S., et al., *Validation of the modified telephone interview for cognitive status (TICS-m) in Hebrew.* International Journal of Geriatric Psychiatry, 2003. **18**(5): p. 381-386.

47. Horan, B., et al., *Development of a new virtual reality test of cognition: assessing the test-retest reliability, convergent and ecological validity of CONVIRT.* BMC Psychology, 2020. **8**(1): p. 61.
