# Supplementary figures and images for: Protocol for a pre-post, mixed-methods feasibility study of the Brain Bootcamp behaviour change intervention to promote healthy brain ageing in older adults
Source: PLoS One. 2022 Nov 29;17(11):e0272517. doi: 10.1371/journal.pone.0272517 (PMC9707764; doi:10.1371/journal.pone.0272517)

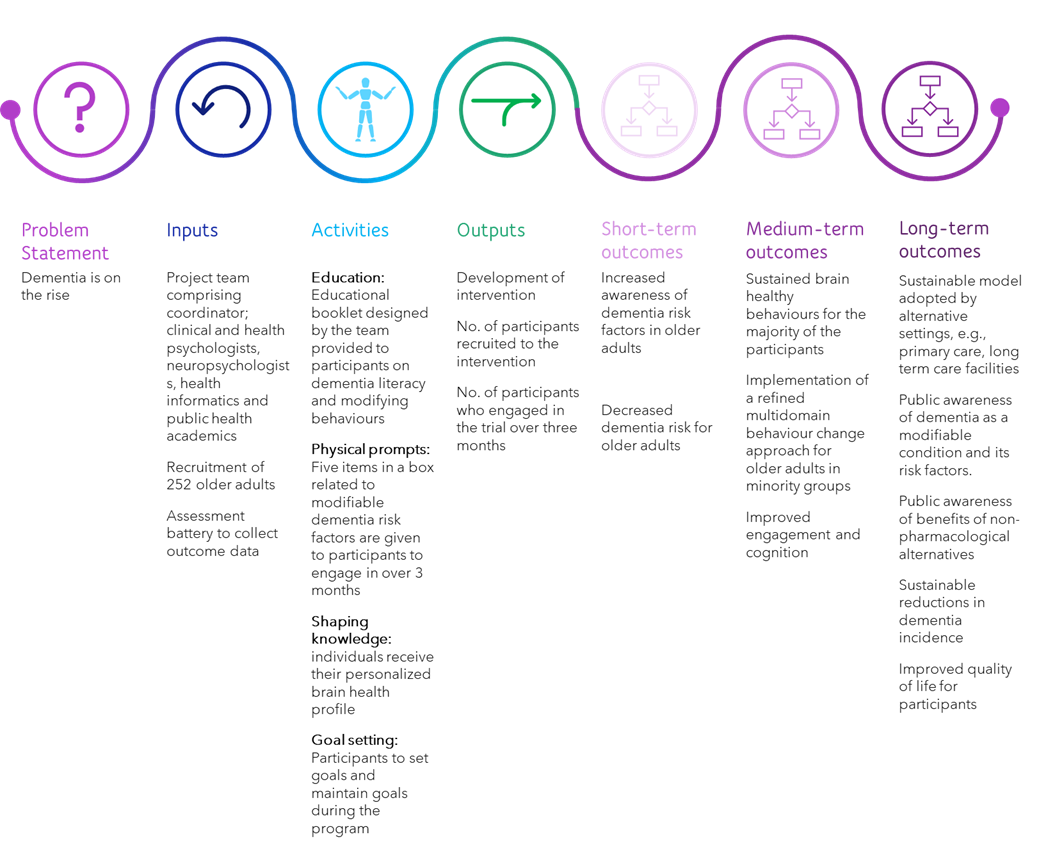

Supplement: S1 Fig — (PNG) [file pone.0272517.s004.png]
